# Conservative Treatments in the Management of Acute Painful Vertebral Compression Fractures: A Systematic Review and Network Meta-Analysis

**DOI:** 10.1001/jamanetworkopen.2024.32041

**Published:** 2024-09-06

**Authors:** Assil-Ramin Alimy, Athanasios D. Anastasilakis, John J. Carey, Stella D’Oronzo, Anda M. Naciu, Julien Paccou, Maria P. Yavropoulou, Willem F. Lems, Tim Rolvien

**Affiliations:** 1 Department of Trauma and Orthopaedic Surgery, University Medical Center Hamburg-Eppendorf, Hamburg, Germany; 2 European Calcified Tissue Society Clinical Practice Action Group, Brussels, Belgium; 3 Department of Endocrinology, 424 General Military Hospital, Thessaloniki, Greece; 4 Department of Rheumatology, Galway University Hospitals, Galway, Ireland; 5 Interdisciplinary Department of Medicine, University of Bari Aldo Moro, Bari, Italy; 6 Unit of Metabolic Bone and Thyroid Diseases, Fondazione Policlinico Universitario Campus Bio-Medico, Rome, Italy; 7 Department of Rheumatology, MABLab ULR 4490, CHU Lille, University Lille, Lille, France; 8 First Department of Propedeutic and Internal Medicine Centre of Expertise for Rare Endocrine Diseases, Medical School National and Kapodistrian University of Athens, Athens, Greece; 9 Department of Rheumatology, Amsterdam University Medical Center, Amsterdam, The Netherlands

## Abstract

**Importance:**

Osteoporotic vertebral compression fractures (VCFs) frequently cause substantial pain and reduced mobility, posing a major health problem. Despite the critical need for effective pain management to restore functionality and improve patient outcomes, the value of various conservative treatments for acute VCF has not been systematically investigated.

**Objective:**

To assess and compare different conservative treatment options in managing acute pain related to VCF.

**Data Sources:**

On May 16, 2023, 4 databases—PubMed, Embase, Scopus, and CINAHL—were searched. In addition, a gray literature search within Scopus and Embase was also conducted.

**Study Selection:**

Included studies were prospective comparative and randomized clinical trials that assessed conservative treatments for acute VCF.

**Data Extraction and Synthesis:**

Data extraction and synthesis were performed by 2 authors according to the Preferred Reporting Items for Systematic Reviews and Meta-Analyses for Network Meta-Analyses recommendations. A frequentist graph-theoretical model and a random-effects model were applied for the meta-analysis.

**Main Outcomes and Measures:**

Primary outcomes were short-term (4 weeks) pain during activity and long-term (latest available follow-up) nonspecified pain in patients with acute VCF.

**Results:**

The study included 20 trials, encompassing 2102 patients, and evaluated various interventions for managing VCF. Calcitonin (standardized mean difference [SMD], −4.86; 95% CI, −6.87 to −2.86) and nonsteroidal anti-inflammatory drugs (NSAIDs; SMD, −3.94; 95% CI, −7.30 to −0.58) were beneficial regarding short-term pain during activity compared with placebo. For long-term nonspecific pain management, bisphosphonates were associated with inferior pain outcomes compared with daily (SMD, 1.21; 95% CI, 0.11 to 2.31) or weekly (SMD, 1.13; 95% CI, 0.05 to 2.21) administration of teriparatide, with no treatment being superior to NSAIDs. The qualitative analysis of adverse events highlighted that typical adverse events associated with these medications were observed.

**Conclusions and Relevance:**

NSAIDs and teriparatide may be the preferred treatment options for pain management in acute osteoporotic VCF. Although calcitonin also proved to be beneficial, its safety profile and potential adverse effects restrict its widespread application. The limited evidence on braces and analgesics underscores the urgent need for future research.

## Key Points

**Question:**

Which conservative treatment option is most beneficial regarding pain-related outcomes in acute osteoporotic vertebral compression fractures (VCFs)?

**Findings:**

Using data from 20 trials involving 2102 patients, this systematic review and network meta-analysis evaluated conservative interventions, including bisphosphonates, calcitonin, teriparatide, nonsteroidal anti-inflammatory drugs (NSAIDs), and braces, for the treatment of acute VCF. The short-term assessment indicated that calcitonin and NSAIDs were associated with decreased pain during activity, and the long-term assessment revealed that teriparatide was associated with lower pain levels compared with bisphosphonates.

**Meaning:**

These findings and currently established treatment strategies highlight the value of NSAIDs and teriparatide in managing pain in acute VCF, while emphasizing the need for further research.

## Teaser

This systematic review and network meta-analysis compares different conservative treatments for managing acute painful vertebral compression fractures.

## Introduction

Osteoporotic vertebral compression fractures (VCFs) are the most common type of osteoporotic fractures and occur primarily in women and men older than 50 years.^1,2^ Although it has been reported that 2 of 3 VCFs remain asymptomatic, those fractures that do present with symptoms often lead to substantial pain, reduced mobility, and quality of life.^3^ Therefore, effective pain management is crucial for restoring functionality and patient outcomes. Regardless of their impact, an optimal and universally accepted reference standard treatment to address pain has not yet been established. Initially, percutaneous surgical techniques gained popularity owing to the promise of effective pain reduction.^4,5,6^ However, various studies^7,8,9^ showed conflicting results concerning the efficacy of these techniques. Because both commonly performed surgical cement augmentation procedures, vertebroplasty and kyphoplasty, have failed to demonstrate consistent benefits in terms of pain relief in previous studies, optimized conservative pain management should be applied first for treating affected patients.

For this reason, the utility of conservative therapies to treat acute painful VCF has been explored in the past.^9,10,11^ These include braces, analgesics such as nonsteroidal anti-inflammatory drugs (NSAIDs) or opioids, but also various antiosteoporotic drugs, including antiresorptives (eg, bisphosphonates, calcitonin, and denosumab) or osteoanabolic agents (eg, teriparatide).^12,13,14,15,16,17,18^ However, the impact of the studies published to date is limited by a lack of comparative analyses, conflicting results, and low statistical power. Given these challenges, there is a critical need for a systematic review and network meta-analysis to thoroughly assess conservative treatment options and provide clinicians with clear, evidence-based guidelines for managing VCF.

Therefore, this systematic review and network meta-analysis aims to assess and compare different conservative treatment options in managing acute pain related to VCF. On the basis of the findings, we provide evidence to guide clinical decision-making and optimize pain management strategies for patients with acute painful VCF.

## Methods

The systematic review and network meta-analysis was conducted according to the guidelines of the Cochrane Collaboration and the Preferred Reporting Items for Systematic Reviews and Meta-Analyses for Network Meta-Analysis recommendations.^19,20^ The review protocol was registered in the PROSPERO database (CRD42023423189).^21^ Informed consent and institutional review board approval were not required because the study does not constitute human participants research, in accordance with 45 CFR §46. The last search was performed on May 16, 2023, in the PubMed, Embase, Scopus, and CINAHL databases. In addition, we also conducted a gray literature search within Scopus and Embase. We further manually reviewed the reference lists of published systematic reviews and included studies. The search strategy was designed according to the Participants, Intervention, Comparator, and Outcome (PICO) model (eTables 1 and 2 in Supplement 1).

### Eligibility Criteria

The inclusion and exclusion criteria were designed in accordance with the PICO model: Eligible studies included randomized clinical trials (RCTs) or prospective comparative studies (PCSs) that examined patients with acute painful VCF.^22^ In these studies, conservative treatment modalities, such as analgesics, antiosteoporotic drugs, other pharmacological interventions, or braces, had to be investigated and compared against no treatment, placebo, or other conservative treatments. To be considered for inclusion, studies were required to assess outcomes related to pain. Furthermore, studies had to be published after January 1996 to align with current medical standards and treatments, particularly with regard to osteoporosis medication.^23,24^ We did not apply further limits regarding the age of patients or the language of publication. Details on data collection and abstraction are presented in the eAppendix in Supplement 1.

### Assessment of Study Quality

Each included study was independently assessed for risk of bias by 2 authors (A.R.A. and T.R.). For RCTs, the assessment was conducted using the second version of the Cochrane tool to assess the risk of bias in randomized trials (RoB2).^25^ For PCSs, the Newcastle-Ottawa Scale was used.^26^ In addition, the Grading of Recommendations, Assessment, Development, and Evaluations (GRADE) approach, tailored for network meta-analysis, was used to appraise the overall certainty of the evidence regarding each outcome (eTable 3 in Supplement 1).^27^ Further details on the assessment of study quality are presented in the eAppendix in Supplement 1.

### Statistical Analysis

The meta-analysis was conducted using the netmeta package in R statistical software version 4.3.3 (R Project for Statistical Computing), using a frequentist graph-theoretical approach.^28^ For continuous outcomes, the standardized mean difference (SMD) was calculated to serve as the effect size in the network meta-analysis. This was performed using the Comprehensive Meta-Analysis software version 4 (Biostat). A random-effects model with inverse variance weighting was implemented to accommodate heterogeneity across studies, given the inherent heterogeneity of treatment protocols, dosages, and durations. To evaluate consistency within the network, the net splitting technique was used, along with the separate indirect from direct evidence method using the back-calculation method.^29^ This method divides network estimates into direct and indirect evidence comparisons, facilitating the identification of inconsistencies in individual comparison estimates. Although a subgroup analysis based on the risk of bias assessment was initially planned, it was deemed unfeasible because most studies were classified with an intermediate risk of bias. Furthermore, although funnel plots were considered for assessing publication bias, this was ultimately not pursued owing to insufficient statistical power, primarily because most subgroups comprised fewer than 10 trials.^19^ Treatment ranking was conducted by using P-scores, which estimate the probability of one treatment being superior to the others on a continuous scale from 0 to 1.^30^

## Results

**Figure 1.**
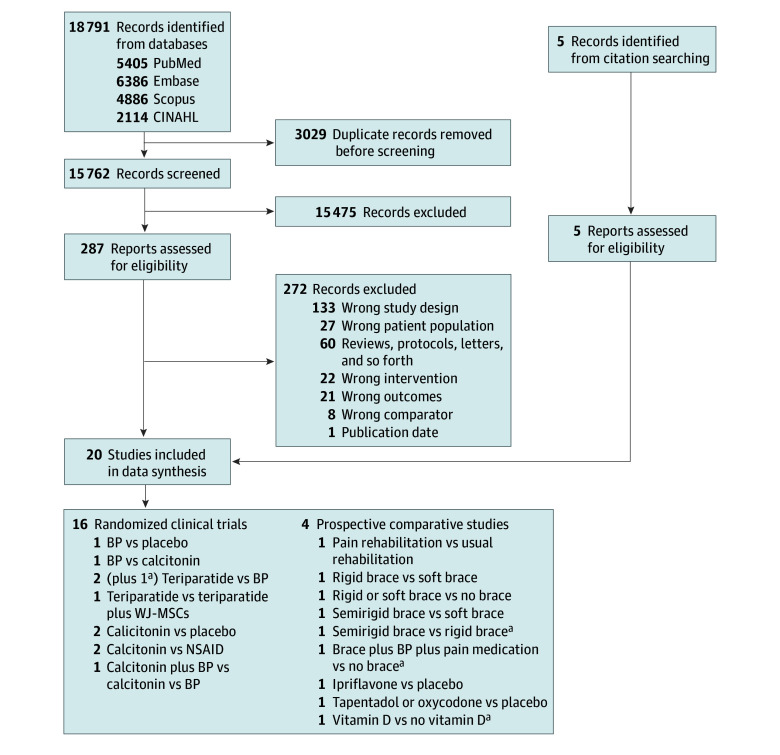
Flowchart of Included Studies BP indicates bisphosphonate; NSAID, nonsteroidal anti-inflammatory drug; and WJ-MSC, Wharton jelly–derived mesenchymal stem cell. ^a^Refers to the 4 prospective comparative studies.

Our search identified 18 791 articles, and after duplicate removal, we reviewed 15 762 titles and abstracts, resulting in 287 full-text articles evaluated for eligibility (eTable 4 in Supplement 1). Moreover, citation searching led to the assessment of an additional 5 studies. This ultimately resulted in the final inclusion of 20 studies in our analysis,^16,31,32,33,34,35,36,37,38,39,40,41,42,43,44,45,46,47,48,49^ including 16 RCTs and 4 prospective comparative studies (Figure 1 and eTable 5 in Supplement 1). These trials included a variety of different types of interventions related to the conservative management of VCF, including bisphosphonates, calcitonin, teriparatide, NSAIDs, opioids, ipriflavone, vitamin D supplementation, pain rehabilitation, and braces. They were conducted across various global regions, including Asia (Japan,^32,33,34,35,36,43,44,45,46,49^ Korea,^37,38^ Bangladesh,^42^ and Hong Kong^42^), Europe (France,^31,39^ Italy,^41^ and Greece^16,40^), and the US.^47^ The majority of the studies were conducted in hospital environments,^16,31,35,36,37,39,40,43,46,48,49^ a few also took place in clinics and private health care facilities,^38,42,44,45^ and 1 study^32^ included both the hospital and clinic setting.

Most included studies were evaluated as having intermediate quality according to the RoB2 criteria (eFigure 1 in Supplement 1). Among these, one study was identified with a low risk of bias and another with a high risk of bias. The included prospective comparative studies were all evaluated as being of good quality (eTable 6 in Supplement 1). A total of 2102 patients with acute VCF were included in this systematic review and network meta-analysis.

### Pain

#### Short-Term Pain During Activity

**Figure 2.**
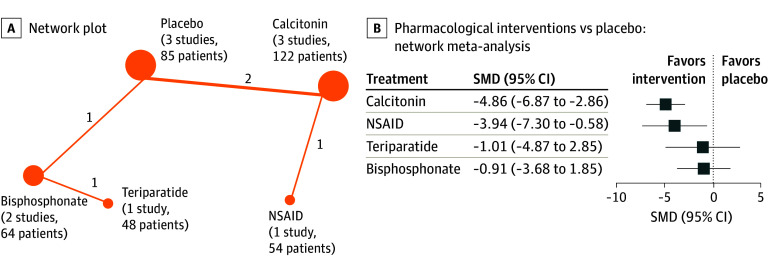
Short-Term Pain During Activity A, Network plot of studies included in network meta-analysis on short-term pain management during activity. Circle size is proportional to the number of trials for each intervention, and the line thickness is proportional to the number of trials comparing the interventions. B, Network meta-analysis for short-term pain management during activity comparing pharmacological interventions with placebo. Data are presented as the standardized mean difference (SMD) and 95% CI for pain during activity. Values below 0 indicate that the treatment mentioned first (before the vs) is favored, whereas values above 0 indicate that the treatment mentioned last (after the vs) is favored. NSAID indicates nonsteroidal anti-inflammatory drug.

Our primary outcome of interest was short-term pain during activity, because early mobilization is one of the most critical objectives in the treatment of VCF. Here, our analysis encompassed a range of treatments, such as calcitonin, bisphosphonates, teriparatide, NSAIDs, and placebo (Figure 2A). Activities included walking, rising from a lying position, and standing. The use of calcitonin was associated with decreased pain compared with bisphosphonates and placebo (SMD, −4.86; 95% CI, −6.87 to −2.86) (Figure 2B and eFigure 2 in Supplement 1). Similarly, NSAIDs demonstrated benefits regarding pain relief compared with placebo (SMD, −3.94; 95% CI, −7.30 to −0.58) (Figure 2B). However, neither teriparatide (SMD, −1.01; 95% CI, −4.87 to 2.85) nor bisphosphonates (SMD, −0.91; 95% CI, −3.68 to 1.85) revealed differences regarding pain relief compared with placebo (Figure 2B). Overall, when ranking treatments for short-term pain management during activity, calcitonin emerged as the most favorable (P-score = 0.92), followed by NSAIDs (P-score = 0.76) (eFigure 3 in Supplement 1). However, it is important to note that most of the results were of low or very low certainty of evidence (eTable 7 in Supplement 1). To further check the robustness of our analysis, we also performed sensitivity analyses by including only walking and rising from a lying position and only walking as activities. Here, similar results were demonstrated (eFigures 4 and 5 in Supplement 1), which strengthens our initial analysis.

#### Long-Term Nonspecified Pain

**Figure 3.**
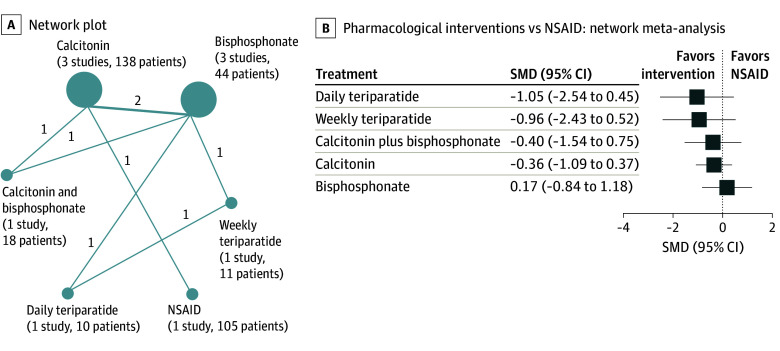
Pharmacological Interventions for Long-Term Pain Management (Nonspecified Pain) A, Network plot of studies included in network meta-analysis of pharmacological interventions regarding long-term pain management. Circle size is proportional to the number of trials for each intervention, and the line thickness is proportional to the number of trials comparing the interventions. B, Network meta-analysis for long-term pain management (nonspecified) comparing pharmacological interventions with nonsteroidal anti-inflammatory drug (NSAID). Data are presented as the standardized mean difference (SMD) and 95% CI for long-term pain outcomes. Values below 0 indicate that the treatment mentioned first (before the vs) is favored, whereas values above 0 indicate that the treatment mentioned last (after the vs) is favored.

Our secondary outcome of interest was longer-term general (ie, nonspecified) pain, aimed at evaluating the association between conservative treatments and patients’ overall pain levels at the latest available follow-up, which was a mean (SD) of 11.5 (9.0) weeks in the included studies. Here, our network consisted of various treatments, including calcitonin, bisphosphonates, teriparatide, and NSAIDs (Figure 3A). Our analysis revealed that both daily (SMD, 1.22; 95% CI, 0.12 to 2.32) and weekly (SMD, 1.13; 95% CI, 0.05 to 2.21) teriparatide had similar superiority over bisphosphonates (eFigure 6 in Supplement 1). However, comparisons with NSAIDs revealed no advantage for either daily (SMD, −1.05; 95% CI, −2.54 to 0.45) or weekly (SMD, −0.96; 95% CI, −2.43 to 0.52) teriparatide (Figure 3B). Similarly, no benefits were observed for the combination of calcitonin and bisphosphonates (SMD, −0.40; 95% CI, −1.54 to 0.75), calcitonin alone (SMD, −0.36; 95% CI, −1.09 to 0.37), or bisphosphonates alone (SMD, 0.17; 95% CI, −0.84 to 1.18) compared with NSAIDs (Figure 3B). When ranking the pharmacological interventions according to their association with long-term pain relief, teriparatide emerged as the most favorable (daily teriparatide, P-score = 0.83; weekly teriparatide, P-score = 0.78), followed by a combination of calcitonin and bisphosphonate (P-score = 0.52) (eFigure 7 in Supplement 1). However, it is important to note that most of the results were of low and very low certainty of evidence (Table).

**Table.** Effect Estimates and Grading of Recommendations, Assessment, Development, and Evaluations Quality Ratings for Comparison of Different Pharmacological Interventions and Braces for Long-Term Pain Management (Nonspecified)

Comparison	Direct evidence		Indirect evidence		Network meta-analysis	
	SMD (95% CI)^a^	Certainty	SMD (95% CI)^a^	Certainty	SMD (95% CI)^a^	Certainty
Bisphosphonate vs calcitonin	0.53 (−0.17 to 1.23)	Low^b^^,^^c^	NA	NA	0.53 (−0.17 to 1.23)	Low^b^^,^^c^
Bisphosphonate vs calcitonin plus bisphosphonate	0.74 (−0.21 to 1.69)	Low^b^^,^^c^	−0.57 (−3.02 to 1.87)	Very low^b^^,^^c^^,^^d^	0.57 (−0.32 to 1.46)	Very low^b^^,^^c^^,^^d^
Bisphosphonate vs daily teriparatide	1.21 (0.11 to 2.31)	Very low^b^^,^^c^^,^^d^	NA	NA	1.22 (0.12 to 2.32)	Very low^b^^,^^c^^,^^d^
Bisphosphonate vs NSAID	NA	NA	0.17 (−0.84 to 1.18)	Very low^b^^,^^c^^,^^d^	0.17 (−0.84 to 1.18)	Very low^b^^,^^c^^,^^d^
Bisphosphonate vs weekly teriparatide	1.14 (0.06 to 2.21)	Very low^b^^,^^c^^,^^d^	NA	NA	1.13 (0.05 to 2.21)	Very low^b^^,^^c^^,^^d^
Calcitonin vs calcitonin plus bisphosphonate	−0.13 (−1.07 to 0.82)	Low^c^^,^^d^	1.21 (−1.28 to 3.71)	Very low^b^^,^^c^^,^^d^	0.04 (−0.84 to 0.92)	Very low^b^^,^^c^^,^^d^
Calcitonin vs daily teriparatide	NA	NA	0.69 (−0.61 to 1.99)	Very low^b^^,^^c^^,^^d^	0.69 (−0.61 to 1.99)	Very low^b^^,^^c^^,^^d^
Calcitonin vs NSAID	−0.36 (−1.09 to 0.37)	Low^b^^,^^c^	NA	NA	−0.36 (−1.09 to 0.37)	Low^b^^,^^c^
Calcitonin vs weekly teriparatide	NA	NA	0.60 (−0.68 to 1.88)	Very low^b^^,^^c^^,^^d^	0.60 (−0.68 to 1.88)	Very low^b^^,^^c^^,^^d^
Calcitonin plus bisphosphonate vs weekly teriparatide	NA	NA	0.56 (−0.83 to 1.96)	Very low^b^^,^^c^^,^^d^	0.56 (−0.83 to 1.96)	Very low^b^^,^^c^^,^^d^
Daily teriparatide vs weekly teriparatide	−0.09 (−1.16 to 0.97)	Very low^b^^,^^c^	NA	NA	−0.09 (−1.15 to 0.98)	Very low^b^^,^^c^
Weekly teriparatide vs NSAID	NA	NA	−0.96 (−2.43 to 0.52)	Very low^b^^,^^c^	−0.96 (−2.43 to 0.52)	Very low^b^^,^^c^
Rigid brace vs no brace	−0.51 (−2.11 to 1.09)	Moderate^c^	1.08 (−2.62 to 4.78)	Very low^b^^,^^c^^,^^d^	−0.26 (−1.73 to 1.21)	Very low^b^^,^^c^^,^^d^
Soft brace vs no brace	−0.28 (−1.89 to 1.32)	Moderate^c^	−1.86 (−5.53 to 1.80)	Very low^b^^,^^c^^,^^d^	−0.54 (−2.01 to 0.93)	Very low^b^^,^^c^^,^^d^
Semirigid brace vs no brace	NA	NA	−1.51 (−3.26 to 0.25)	Very low^b^^,^^c^^,^^d^	−1.51 (−3.26 to 0.25)	Very low^b^^,^^c^^,^^d^
Rigid brace vs soft brace	−0.09 (−1.17 to 1.00)	Low^b^^,^^c^	1.72 (−0.45 to 3.88)	Very low^b^^,^^c^^,^^d^	0.28 (−0.69 to 1.25)	Very low^b^^,^^c^^,^^d^
Rigid brace vs semirigid brace	1.93 (0.42 to 3.44)	Very low^d^	0.16 (−1.73 to 2.06)	Very low^b^^,^^c^^,^^d^	1.25 (0.07 to 2.43)	Very low^b^^,^^c^^,^^d^
Soft brace vs semirigid brace	0.24 (−1.31 to 1.79)	Very low^b^^,^^c^^,^^d^	2.01 (0.15 to 3.87)	Very low^b^^,^^c^^,^^d^	0.97 (−0.22 to 2.16)	Very low^b^^,^^c^^,^^d^

Abbreviations: NA, not applicable; NSAID, nonsteroidal anti-inflammatory drug; SMD, standardized mean difference.

^a^ Data are for nonspecified pain at the latest available follow-up. Values below 0 indicate that the treatment mentioned first (before the vs) is favored, whereas values above 0 indicate that the treatment mentioned (after the vs) is favored.

^b^ Refers to indirectness.

^c^ Refers to imprecision.

^d^ Refers to risk of bias.

**Figure 4.**
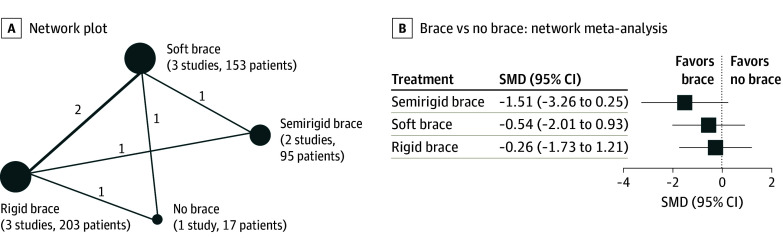
Braces for Long-Term Pain Management (Nonspecified Pain) A, Network plot of studies included in network meta-analysis of brace treatment regarding long-term pain management. Circle size is proportional to the number of trials for each intervention, and the line thickness is proportional to the number of trials comparing the interventions. B, Network meta-analysis for long-term pain management (nonspecified) comparing the treatment with various braces with no brace treatment. Data are presented as the standardized mean difference (SMD) and 95% CI for long-term pain. Values below 0 indicate that the treatment mentioned first (before the vs) is favored, whereas values above 0 indicate that the treatment mentioned last (after the vs) is favored regarding pain outcomes.

In addition to pharmacological interventions, we evaluated braces for their association with pain relief for VCF. Here, our network consisted of 4 studies that included rigid, soft, and semirigid active braces and no brace treatment with a mean (SD) latest available follow-up of 22.00 (19.18) weeks (Figure 4A). Regarding the use of braces for long-term nonspecified pain management, our findings indicated no benefit for semirigid braces (SMD, −1.51; 95% CI, −3.26 to 0.25), soft braces (SMD, −0.54; 95% CI, −2.01 to 0.93), or rigid braces (SMD, −0.26; 95% CI, −1.73 to 1.21) compared with no brace (Figure 4B and eFigure 8 in Supplement 1). For the use of braces, despite not showing benefits over no brace use, a comparative ranking was performed. Here, the semirigid brace was ranked highest (P-score = 0.96), followed by the soft brace (P-score = 0.51) (eFigure 9 in Supplement 1). However, again, most results were of low or very low certainty of evidence (Table).

### Adverse Events

Adverse events were assessed qualitatively, because pooling of adverse events of varying severity was deemed inappropriate. Our qualitative analysis revealed no apparent differences between the different interventions but highlighted that typical adverse events associated with these medications were frequently observed (eTable 8 in Supplement 1). For instance, gastrointestinal complaints were often reported with calcitonin use; in particular, upper gastrointestinal disorders were related to NSAIDs in 1 patient.^16,32,39^

## Discussion

Osteoporotic VCF is one of the leading causes of pain and disability worldwide. Despite the major impact of these fractures, a definitive pain management treatment standard has yet to be established, contributing to uncertainty among physicians and patients. Although surgical options like kyphoplasty and vertebroplasty are available, their value compared with conservative or sham treatments is still debated.^7,8,9,11,50^ Consequently, conservative management has emerged as a viable option to treat acute pain for many patients, dictating the need for further investigation into its applicability, benefits, and risks. However, to date, there has been no conclusive answer regarding different conservative treatment modalities for VCF and their comparative analysis of pain outcomes.

In this systematic review and network meta-analysis, we focused primarily on short-term pain during activity as the main outcome parameter because of its critical importance from a clinical perspective. Avoiding prolonged bed rest can reduce the risks associated with immobility, such as worsening osteopenia, musculoskeletal function, and neurological compromise.^51^ Effectively managing pain during activity and, thus, facilitating early mobilization is essential to improving patient care and was, therefore, one primary focus of our study.

Our findings revealed that calcitonin was associated with superior outcomes in managing particularly short-term pain across activity. This suggests the potential for calcitonin as a pharmacological intervention in managing acute pain in VCF, although further research is warranted for a comprehensive understanding. In this context, it is essential to consider the adverse effects of calcitonin, such as nausea, vomiting, and headaches, in the clinical decision-making process, which were also observed in our qualitative analysis of adverse events. Moreover, the European Medicines Agency’s decision to revoke intranasal calcitonin-containing product’s approval for osteoporosis treatment, because of the associated long-term cancer risk, underscores the need for caution.^52,53^ Nevertheless, calcitonin remains approved for preventing acute bone loss in cases of sudden immobilization, like recent osteoporotic fractures, although its limited availability also restrains its use in this setting. Given our results, calcitonin should be further evaluated in terms of its potential use and safety profile in acute VCF, ensuring that a comprehensive risk-benefit analysis informs treatment decisions.

Given the favorable results of calcitonin in our study, it appeared crucial to examine the performance of more commonly used antiosteoporotic drugs, such as antiresorptive and osteoanabolic agents. It can be speculated that short-term pain-related outcomes are primarily associated with the direct reduction of pain, whereas long-term outcomes are more likely influenced by the decrease in the incidence of new VCF. Surprisingly, bisphosphonates were not associated with decreased pain regarding both short-term pain during activity and long-term nonspecified pain. This was the case for both oral and intravenous bisphosphonates, as our analysis incorporated various forms of bisphosphonates, including pamidronate (intravenous),^31,39^ alendronate (oral),^34,43^ risedronate (oral),^43,46^ and minodronic acid hydrate (oral).^44^ Therefore, our data suggest that all available bisphosphonates have similar overall results for VCF pain.

Importantly, both daily and weekly administration of teriparatide were found to be more beneficial than bisphosphonates regarding long-term nonspecified pain. It has been proposed that teriparatide might exert its effects through anabolic mechanisms that promote fracture healing.^43,54^ In addition, teriparatide could directly alleviate pain by decreasing proinflammatory cytokines.^55^ Its superior results regarding long-term pain relief compared with bisphosphonates, which function primarily through antiresorptive mechanisms, might derive from its anabolic properties. The analgesic properties of bisphosphonates have been proposed to result from osteoclast inactivation and reduction of the acidic environment, in contrast to teriparatide, which promotes bone formation and may, therefore, be better tailored for VCF than bisphosphonates.^56^

Our results highlight a notable gap regarding evidence on analgesics for acute painful VCF. Our extensive search identified only 2 studies evaluating NSAIDs^32,45^ and 1 study^47^ assessing the role of opioids. Notably, NSAIDs were associated with less pain during activity compared with placebo, suggesting an important role for NSAIDs in acute short-term pain control of VCF. The study^47^ examining opioids, specifically tapentadol and oxycodone, failed to find any difference compared with placebo but was prematurely terminated, limiting its capacity to detect meaningful differences at all. This indicates a pressing need for further research to comprehensively evaluate the role of both nonopioid and opioid analgesics in managing pain for patients with acute VCF.

In evaluating braces, we found no benefit in long-term pain management, aligning with other studies.^57^ However, it is important to interpret these results with caution, because pain in VCF may last for only 6 to 8 weeks.^58,59^ Therefore, although braces may be appropriate and beneficial for initial immobilization, our findings do not support long-term benefits from their use. Nevertheless, additional high-quality RCTs are necessary to enable a comprehensive assessment of braces, particularly regarding their short-term outcomes.

Our initial motivation to perform this meta-analysis was based on the observation of one of the authors (W.F.L.), while preparing a presentation for the European Calcified Tissue Society 2023 annual meeting, that there was surprisingly little evidence on conservative treatment for the management of acute painful VCF. Above all, the high burden of affected patients, as well as the inconsistent results of surgical procedures such as vertebroplasty and kyphoplasty, should be reason enough to investigate the outcomes related to conservative treatment modalities thoroughly. The results of this systematic review and network meta-analysis may indicate a favorable role of teriparatide, which also shows excellent results in increasing vertebral bone mineral density and reducing the risk of vertebral fractures.^60^ This finding is critical because it shows that an antiosteoporotic drug may also have a pain-reducing effect, which supports its use promptly after a fracture occurs. However, we were surprised by the poor evidence for other commonly used antiosteoporotic drugs, such as denosumab, calling for the need to conduct high-quality RCTs on the role of denosumab in pain management after an acute VCF. Furthermore, on the basis of the promising data on teriparatide, the outcomes related to romosozumab, the most recently approved osteoporosis medication with a dual osteoanabolic-antiresorptive effect, should also be evaluated in this context.^61^ In our opinion, the lack of benefits of braces regarding long-term pain relief is clinically highly relevant, and more high-quality studies are needed, particularly evaluating their role in short-term pain relief. Another critical point is that there were only 3 studies^32,45,47^ on analgesics, which is why future studies should specifically evaluate the outcomes of targeted pain protocols using analgesics such as NSAIDs and opioids. We would like to motivate both researchers and funding agencies to conduct and support studies on conservative pain management in patients with acute VCF, using controlled study designs that will evaluate specific treatment modalities against each other or placebo. The development of evidence-based treatment guidelines depends on more high-quality clinical research, especially RCTs.

### Limitations

Our study has several limitations. The included studies exhibited high clinical heterogeneity. For instance, the studies used various forms of calcitonin (salmon, eel, and synthetic human derivatives) and bisphosphonates (oral, intravenous, and various preparations). Although this diversity broadens our analysis, providing a comprehensive overview of the overall value regarding pain relief, it also increases heterogeneity, potentially hindering our ability to detect true differences between treatments. Furthermore, we included both RCTs and PCSs, which introduces variability in study quality and inevitably increases the risk of bias. However, the PCSs included were all of high quality, contributing to a more robust and comprehensive analysis. Another limitation of our study is the potential for confounders owing to varying treatment protocols among the studies. Differences in patients receiving prior antiosteoporotic therapy and the permitted use of NSAIDs or the additional application of braces for early immobilization also contribute to heterogeneity. Although these practices are ethically justified and reflect real-world clinical settings, they may limit the comparability of study outcomes. In addition, our primary outcome, pain during activity, presents an inherent limitation, because activities such as walking, rising from a lying position, and standing were not uniformly defined but were pooled together. This also leads to increased heterogeneity. However, we have conducted sensitivity analyses regarding this outcome to strengthen the robustness of our findings and mitigate the impact of different activities pooled together. A further limitation of our study is that the generalizability and applicability of our results is certainly limited, which is reflected by our GRADE assessment, which revealed low to very low certainty of evidence for most of our results but emphasizes, therefore, once again the need for more high-quality research. In addition, our review lacks not only an analysis of functional outcomes but also quality-of-life outcomes because they were not consistently assessed and reported across studies. Therefore, future studies should include these outcomes to allow for a more comprehensive pooling of data in the future.

Furthermore, our results on braces, as reported in our findings, should be interpreted with caution. The data regarding braces often rely on patient compliance, which can be ensured only to a certain extent. Despite their frequent use in clinical practice and positive anecdotal evidence, our study underscores the need for more research to provide more definitive evidence that can guide clinical practice and confirm or challenge the clinical value of braces.

## Conclusions

Taken together, our study provides a comprehensive analysis of various conservative treatments for acute VCF. We identified that calcitonin, in its various forms, and teriparatide stand out for their ability in managing both short-term and long-term pain management associated with these fractures. Given the limitations arising from the included studies’ quality and heterogeneity and the lack of sufficient studies, especially on analgesics, our analysis calls for further high-quality RCTs to support clinical decision-making.

## Notes

Supplement 1. **eAppendix**. Supplemental methods **eTable 1.** Search strategy **eTable 2.** Inclusion criteria **eTable 3.** Framework for the GRADE assessment **eTable 4.** List of excluded studies **eTable 5.** Characteristics of the included studies **eTable 6.** Study quality of the included prospective, comparative studies **eTable 7.** Estimates of effects and GRADE quality ratings for comparison of different pharmacological interventions for short-term pain during activity **eTable 8.** Adverse events of the included studies **eFigure 1.** Risk of bias assessment **eFigure 2.** Node splitting: Short-term pain during activity **eFigure 3.** P-score ranking: Short-term pain during activity **eFigure 4.** Sensitivity analyses: Short-term pain during activity (walking and rising up) **eFigure 5.** Sensitivity analyses: Short-term pain during activity (only walking) **eFigure 6.** Node splitting: Pharmacological interventions (long-term pain) **eFigure 7.** P-score ranking: Pharmacological interventions for long-term pain **eFigure 8.** Node splitting: Braces (long-term pain) **eFigure 9.** P-score ranking: Braces for long-term pain
eReferences

Supplement 2.
Data Sharing Statement
