# Supplementary material for: Conservative Treatments in the Management of Acute Painful Vertebral Compression Fractures: A Systematic Review and Network Meta-Analysis
Source: JAMA Netw Open. 2024 Sep 6;7(9):e2432041. doi: 10.1001/jamanetworkopen.2024.32041 (PMC11380106; doi:10.1001/jamanetworkopen.2024.32041)
Supplement: Supplement 1. — eAppendix. Supplemental methods eTable 1. Search strategy eTable 2. Inclusion criteria eTable 3. Framework for the GRADE assessment eTable 4. List of excluded studies eTable 5. Characteristics of the included studies eTable 6. Study quality of the included prospective, comparative studies eTable 7. Estimates of effects and GRADE quality ratings for comparison of different pharmacological interventions for short-term pain during activity eTable 8. Adverse events of the included studies eFigure 1. Risk of bias assessment eFigure 2. Node splitting: Short-term pain during activity eFigure 3. P-score ranking: Short-term pain during activity eFigure 4. Sensitivity analyses: Short-term pain during activity (walking and rising up) eFigure 5. Sensitivity analyses: Short-term pain during activity (only walking) eFigure 6. Node splitting: Pharmacological interventions (long-term pain) eFigure 7. P-score ranking: Pharmacological interventions for long-term pain eFigure 8. Node splitting: Braces (long-term pain) eFigure 9. P-score ranking: Braces for long-term pain eReferences [file jamanetwopen-e2432041-s001.pdf]

Supplemental Online Content

Alimy AR, Anastasilakis AD, Carey JJ, et al. Conservative treatments in the management of acute painful vertebral compression fractures: a systematic review and network meta-analysis. *JAMA Netw Open*. 2024;7(9):e2432041.
doi:10.1001/jamanetworkopen.2024.32041

eAppendix. Supplemental Methods
eTable 1. Search strategy
eTable 2. Inclusion criteria
eTable 3. Framework for the GRADE assessment
eTable 4. List of excluded studies
eTable 5. Characteristics of the included studies
eTable 6. Study quality of the included prospective, comparative studies
eTable 7. Estimates of effects and GRADE quality ratings for comparison of different pharmacological interventions for short-term pain during activity
eTable 8. Adverse events of the included studies
eFigure 1. Risk of bias assessment
eFigure 2. Node splitting: Short-term pain during activity
eFigure 3. P-score ranking: Short-term pain during activity
eFigure 4. Sensitivity analyses: Short-term pain during activity (walking and rising up)
eFigure 5. Sensitivity analyses: Short-term pain during activity (only walking)
eFigure 6. Node splitting: Pharmacological interventions (long-term pain)
eFigure 7. P-score ranking: Pharmacological interventions for long-term pain
eFigure 8. Node splitting: Braces (long-term pain)
eFigure 9. P-score ranking: Braces for long-term pain
eReferences

This supplemental material has been provided by the authors to give readers additional information about their work.

© 2024 Alimy AR et al. *JAMA Network Open.*

eAppendix. Supplemental Methods

Data collection and abstraction
Each database was searched independently, and the resulting datasets were exported to EndNote^{TM} version 21.2 (Clarivate, Philadelphia, PA, USA) for deduplication. After deduplication, the datasets were transferred to Covidence^{TM} (Veritas Health Innovation, Melbourne, Australia) for further processing. Initially, two authors (ARA and TR) screened all titles and abstracts, and any disagreements were resolved through discussion. Subsequently, full texts were screened by two authors (ARA and TR). Data extraction was performed using a standardized proforma in Covidence by two authors (ARA and TR). For the qualitative data synthesis, information such as the period during which the study was conducted, the year of publication, the country of the study, and demographic details of all participants, along with the treatments administered in each study arm, including individual doses, and complications as well as adverse events was extracted. For quantitative data synthesis, summary estimates, including means, standard deviations (SDs), and sample sizes, were extracted for pain during activity at the 4-week follow-up (short-term) and further for non-specified pain at the latest available follow-up (long-term). If means and SDs were not provided, medians and ranges were converted to means and SDs.[1] If data were only presented in graphs, extraction was performed using PlotDigitizer version 3.1.5.[2]

Assessment of Study Quality
The Cochrane RoB2 tool facilitates the evaluation of the risk of bias across six distinct domains. A study was considered to have a low risk of bias only if all domains were assessed as low risk. If any domain was rated as having "some concerns," the study was classified as having an intermediate risk of bias. Studies were deemed to have a high risk of bias if they exhibited a high risk in at least two domains. The evaluation of the individual domains for each study is visualized by a risk of bias graph and a risk of bias summary figure.
For prospective comparative studies, the Newcastle-Ottawa Scale (NOS) was utilized to assess quality across three key domains: the selection of cohorts, comparability of groups, and the assessment of outcomes. Based on these evaluations, studies were categorized into “good”. “fair”, or “poor” quality ratings.[3]

© 2024 Alimy AR et al. *JAMA Network Open.*

eTable 1: Search strategy

Search strategy for the PubMed database.

PubMed	No	Query
	#1	“("Spinal Fractures"[Mesh] OR "Osteoporotic Fractures"[Mesh] OR "Fractures, Compression" [Mesh] OR "Osteoporosis[Mesh]" OR "osteoporotic fracture"[Title/Abstract] OR "vertebr*"[Title/Abstract] OR "spinal"[Title/Abstract] OR "compression fractures"[Title/Abstract] OR "bone density"[Title/Abstract] OR "osteopor*"[Title/Abstract] "kyphotic deformity"[Title/Abstract] OR "fragility fractures"[Title/Abstract] OR "spine fractures"[Title/Abstract] OR "compression fractures"[Title/Abstract] OR "vertebral collapse "[Title/Abstract] OR "VCF"[Title/Abstract] OR "degenerative spinal"[Title/Abstract] OR "OVCF*"[Title/Abstract] OR "osteoporotic vertebral compression fractures" [Title/Abstract])
	#2	("placebos"[Mesh] OR "sham treatment"[Title/Abstract] OR "vertebroplasty"[Mesh] OR "kyphoplasty"[Mesh] OR "vertebral augmentation"[Title/Abstract] OR "vertebral reconstruction"[Title/Abstract] OR "vertebral stabilization"[Title/Abstract] OR "vertebral fixation"[Title/Abstract] OR "spinal fusion"[Mesh] OR "Cementoplasty"[Mesh] OR "Surgical Procedures, Operative"[Mesh] OR "spinal surgery"[Title/Abstract] OR "surgical treatment"[Title/Abstract] OR "kyphoplasty"[Title/Abstract] OR "balloon kyphoplasty"[Title/Abstract] OR "percutaneous vertebroplasty"[Title/Abstract] OR "vertebral augmentation"[Title/Abstract] OR "cement"[Title/Abstract] OR "vertebroplasty"[Title/Abstract] OR "PVP"[Title/Abstract] OR "CT"[Title/Abstract] OR "PVA"[Title/Abstract] OR "PKP"[Title/Abstract] OR "NB"[Title/Abstract] OR "conservative"[Title/Abstract] OR "CT"[Title/Abstract] OR "non-surgical treatment"[Title/Abstract] OR "non-operative treatment"[Title/Abstract] OR "nonsurgery "[Title/Abstract] "non-operative management"[Title/Abstract] OR "non-operative therapy"[Title/Abstract] OR "medical management"[Title/Abstract] OR "pharmacological treatment"[Title/Abstract] OR "drug treatment"[Title/Abstract] OR "medication"[Title/Abstract] OR "non-pharmacological treatment"[Title/Abstract] OR "opoid*" [Title/Abstract] OR "tapentadol" [Title/Abstract] OR "oxycodone" [Title/Abstract] OR "fentanyl" [Title/Abstract] OR "calcitonin"[Title/Abstract] OR "bisphosp*"[Title/Abstract] OR "braces" [Title/Abstract] OR "physi*" [Title/Abstract] OR "excerc*" [Title/Abstract] OR "analge*" [Title/Abstract] OR "percutaneous" [Title/Abstract] OR "pain" [Title/Abstract] OR "OPM" [Title/Abstract])

© 2024 Alimy AR et al. *JAMA Network Open.*

eTable 2: Inclusion criteria

Inclusion criteria based on the Population, Intervention, Comparator and Outcome (PICO) model.

Domains	Inclusion criteria
Population	Adults (>18 years old) with single or multiple, acute, painful vertebral fractures in the context of osteoporosis
Intervention/Comparator	Conservative treatment compared to other conservative interventions or placebos
Reported Outcomes	Pain and quality of life
Study Design	Randomized controlled trials and prospective comparative studies

© 2024 Alimy AR et al. *JAMA Network Open.*

eTable 3: Framework for the GRADE assessment.

GRADE assessment	
Starting point	The initial quality rating depends on the study design. For randomized trials, the quality starts as “high”, whereas for observational studies, it starts as “low”.
Risk of bias	The quality was downgraded if the RoB 2 (Risk of Bias 2) assessment indicated a high risk of bias in Domain 1.
Imprecision	The quality was downgraded if the confidence interval crossed zero or the small effect size threshold based on Cohen’s d (0.2).
Indirectness	The quality was downgraded if there were notable differences in population characteristics, treatment modalities (e.g., different medications, forms of application), or outcome assessment methods.

© 2024 Alimy AR et al. *JAMA Network Open.*

eTable 4: List of excluded studies

Reason	No.	Authors	Year	Title
**Publication date**	1	Lyritis, G. P.; Tsakalakos, N.; Magiasis, B.; Karachalios, T.; Yiatzides, A.; Tsekoura, M.	1991	Analgesic effect of salmon calcitonin in osteoporotic vertebral fractures: a double-blind placebo-controlled clinical study
**Reviews, Protocols, Letters etc.**	1	Barr, J.D.	2016	Randomized controlled trial of vertebroplasty versus kyphoplasty in the treatment of vertebral compression fractures
	2	Boszczyk, B.	2015	Percutaneous vertebroplasty does not reduce pain and disability from osteoporotic vertebral compression fracture
	3	Briggs, Mimi C; Popeo, Dennis M; Pasculli, Rosa M; Bryson, Ethan O; Kellner, Charles H	2012	Safe resumption of electroconvulsive therapy (ECT) after vertebroplasty.
	4	Buchbinder, R.; Busija, L.	2019	Why we should stop performing vertebroplasties for osteoporotic spinal fractures
	5	Buchbinder, R.; Osborne, R.H.; Kallmes, D.	2009	Vertebroplasty appears no better than placebo for painful osteoporotic spinal fractures, and has potential to cause harm
	6	Cook, E.; Scantlebury, A.; Booth, A.; Turner, E.; Ranganathan, A.; Khan, A.; Ahuja, S.; May, P.; Rangan, A.; Roche, J.; Coleman, E.; Hilton, C.; Corbacho, B.; Hewitt, C.; Adamson, J.; Torgerson, D.; McDaid, C.	2021	Surgery versus conservative management of stable thoracolumbar fracture: The presto feasibility RCT
	7	Diamond, T.; Clark, W.; Bird, P.; Gonski, P.	2019	Percutaneous Vertebroplasty for Acute Painful Osteoporotic Vertebral Fractures,ÄîBenefits Shown in VAPOUR Trial Masked When Pooled With Other Clinical Trials
	8	Dudeney, S.; Lieberman, I.; Cotten, A.; Boutry, N.; Cortet, B.	2000	Percutaneous vertebroplasty in the treatment of osteoporotic vertebral compression fractures: An open prospective study (letters)
	9	Gray, L. A.; Jarvik, J. G.; Heagerty, P. J.; Hollingworth, W.; Stout, L.; Comstock, B. A.; Turner, J. A.; Kallmes, D. F.	2007	INvestigational Vertebroplasty Efficacy and Safety Trial (INVEST): a randomized controlled trial of percutaneous vertebroplasty
	10	Graziotti PJ; Graziotti, Paul J	2010	Vertebroplasty appears no better than placebo for painful osteoporotic spinal fractures, and has potential to cause harm.
	11	Heini, P.F.	2010	Vertebroplasty: an update: value of percutaneous cement augmentation after randomized, placebo-controlled trials.
	12	Hoffmeister, Ellen	2011	Researchers find vertebroplasty not superior to placebo for recent-onset fracture or severe back pain.
	13	Klazen, C.A.H.; Lohle, P.N.M.; DeVries, J.	2010	Erratum: Vertebroplasty versus conservative treatment in acute osteoporotic vertebral compression fractures (Vertos II): An open-label randomised trial
	14	Levy, Jason R; Beall, Douglas P	2014	Vertebral augmentation for symptomatic compression fractures is supported by randomized clinical trials.
	15	Piazzolla, A.; Bizzoca, D.; Balsano, M.; Moretti, B.	2017	Capacitive biophysical stimulation in the treatment of vertebral compression fractures
	16	Pile KD; Pile, Kevin D	2010	Vertebroplasty appears no better than placebo for painful osteoporotic spinal fractures, and has potential to cause harm.
	17	Steurer, J.	2016	Vertebroplasty alleviates pain in acute vertebral body fractures more substantially than placebo
	18	Firanescu, C. E.; de Vries, J.; Lodder, P.; Venmans, A.; Schoemaker, M. C.; Smeets, A. J.; Donga, E.; Juttmann, J. R.; Klazen, C. A. H.; Elgersma, O. E. H.; Jansen, F. H.; Tielbeek, A. V.; Boukrab, I.; Schonenberg, K.; van Rooij, W. J. J.; Hirsch, J. A.; Lohle, P. N. M.	2018	Erratum: Vertebroplasty versus sham procedure for painful acute osteoporotic vertebral compression fractures (VERTOS IV): randomised sham controlled clinical trial

© 2024 Alimy AR et al. *JAMA Network Open.*

	19	Kallmes, D. F.; Comstock, B. A.; Heagerty, P. J.; Turner, J. A.; Wilson, D. J.; Diamond, T. H.; Edwards, R.; Gray, L. A.; Stout, L.; Owen, S.; Hollingworth, W.; Ghdoke, B.; Annesley-Williams, D. J.; Ralston, S. H.; Jarvik, J. G.	2012	A randomized trial of vertebroplasty for osteoporotic spinal fractures
	20	Takano, Y.; Kubota, M.; Takebayashi, K.; Kihara, K.; Yuzurihara, M.; Tachibana, S.; Kawamata, T.	2018	Availability of Early Balloon Kyphoplasty for the Treatment of Osteoporotic Compression Fractures
	21	Buchbinder R; Kallmes D; Glasziou P	2010	Vertebroplasty versus conservative treatment for vertebral fractures
	22	Albers, S.L.; Latchaw, R.E.	2013	The effects of randomized controlled trials on vertebroplasty and kyphoplasty: A square peg in a round hole
	23	Barker, K. L.; Room, J.; Knight, R.; Hannink, E.; Newman, M.	2022	Physiotherapy exercise rehabilitation with tailored exercise adherence support for people with osteoporosis and vertebral fractures: protocol for a randomised controlled trial - the OsteoPorosis Tailored exercise adherence INtervention (OPTIN) study
	24	Blasco, J.; Garcia, A.; Manzanera, L.S.R.; MacHo, J.M.; Peris, P.; Jaume, P.; Criado, E.	2010	Randomized trial comparing vertebroplasty and conservative treatment analyzing pain relief and quality of life on the long term basis
	25	Buchbinder, R.; Osborne, R.H.; Ebeling, P.R.; Wark, J.D.; Mitchell, P.; Wriedt, C.J.; Wengier, L.; Connell, D.; Graves, S.E.; Staples, M.P.; Murphy, B.	2008	Efficacy and safety of vertebroplasty for treatment of painful osteoporotic vertebral fractures: A randomised controlled trial
	26	Buchbinder, R.; Osborne, R.H.; Wark, J.D.; Mitchell, P.; Wreidt, C.; Graves, S.; Staples, M.; Murphy, B.	2009	Efficacy and safety of vertebroplasty for treatment of painful osteoporotic vertebral fractures: A randomised double-blind placebo-controlled trial
	27	Clark, W.; Bird, P.; Diamond, T.; Gonski, P.	2015	Vertebroplasty for acute painful osteoporotic fractures (VAPOUR): Study protocol for a randomized controlled trial
	28	Clark, W.A.	2016	Update on the VAPOR study
	29	Cummings, S.R.; Wardlaw, D.; Van Meirhaeghe, J.; Bastian, L.; Tillman, J.B.; Ranstam, J.; Eastell, R.; Shabe, P.; Talmadge, K.; Boonen, S.	2009	A randomized trial of balloon kyphoplasty and nonsurgical care for acute vertebral compression fracture
	30	Firanescu, C.; Lohle, P. N.; de Vries, J.; Klazen, C. A.; Juttmann, J. R.; Clark, W.; van Rooij, W. J.	2011	A randomised sham controlled trial of vertebroplasty for painful acute osteoporotic vertebral fractures (VERTOS IV)
	31	Javier, P.A.; Patricia, A.G.; Felisa, S.-M.D.; Lorenzo, Z.G.; Sergio, M.B.; Elisa, S.B.; Enrique, I.N.	2013	Two-year outcome in a prospective cohort of patients with vertebral fractures
	32	Klazen, C.; Lohle, P.; Jansen, F.; Schoemaker, M.; Elgersma, O.; Van Everdingen, K.; Fransen, H.; Lo, T.; Tielbeek, A.; Mali, W.	2009	1-year results of the VERTOS II trial: Vertebroplasty versus conservative therapy
	33	Klazen, C.A.; Venmans, A.; Van Rooij, W.-J.J.; Jansen, F.H.; Tielbeek, A.V.; Schoemaker, M.C.; Van Everdingen, K.J.; Fransen, H.; Elgersma, O.E.; Lohle, P.N.M.; Mali, W.P.	2010	Percutaneous vertebroplasty is not a risk factor for new osteoporotic compression fractures: Results from VERTOS II
	34	Lee, H. J.; Seo, J. C.; Park, S. H.; Kwak, M. A.; Shin, I. H.; Min, B. M.; Cho, M. S.; Roh, W. S.; Jung, J. Y.	2015	Acupuncture in patients with a vertebral compression fracture: a protocol for a randomized, controlled, pilot clinical trial
	35	Lohle, P.; Venmans, A.	2019	Abstract No. 299 Percutaneous vertebroplasty is not a risk factor for new fractures and protects against further height loss: results from VERTOS IV
	36	Lohle, P.N.M.; Firanescu, C.E.; De Vries, J.	2016	Update on VERTOS IV
	37	Martinez-Ferrer, √Ä.; Peris, P.; Blasco, J.; Carrasco, J.L.; Monegal, A.; Pom√©s, J.; Gua√±abens, N.	2011	Effect of vertebroplasty on the quality of life of patients with pain related to osteoporotic vertebral fractures: Preliminary results of a randomized trial

© 2024 Alimy AR et al. *JAMA Network Open.*

	38	Martinez-Ferrer, A.; Peris, P.; Blasco, J.; Carrasco, J.-L.; Monegal, A.; Poms, J.; Guanabens, N.	2010	Effect of Vertebroplasty on the quality of life of patients with pain related to osteoporotic vertebral fractures. Preliminary results of a randomized trial
	39	Sciamanna, C.; Ballentine, N. H.; Bopp, M.; Brach, J. S.; Chinchilli, V. M.; Ciccolo, J. T.; Conroy, M. B.; Fisher, A.; Fox, E. J.; Greenspan, S. L.; Jan De Beur Suzanne, M.; Kearcher, K.; Kraschnewski, J. L.; McTigue, K. M.; McAuley, E.; Morone, N. E.; Paranjape, A.; Rodriguez-Colon, S.; Rosenzweig, A.; Smyth, J. M.; Stewart, K. J.; Stuckey, H. L.	2018	Working to Increase Stability through Exercise (WISE): Study protocol for a pragmatic randomized controlled trial of a coached exercise program to reduce serious fall-related injuries
	40	Spiegl, U.; Schenk, P.; Schnake, K.J.; Ullrich, B.W.; Osterhoff, G.; Scheyerer, M.J.; Schmeiser, G.; Katscher, S.; Der Dkou, A.O.F.	2022	Treatment and outcome of osteoporotic thoracolumbar vertebral body fractures with deformation of both endplates with or without posterior wall involvement (OF 4) - short-term results from the prospective EOFTT multicenter study
	41	Wardlaw, D.; Cummings, S.; Van Meirhaeghe, J.; Bastian, L.; Tillman, J.; Ranstam, J.; Eastell, R.; Shabe, P.; Talmadge, K.; Boonen, S.	2009	A randomized trial of balloon kyphoplasty and nonsurgical care for acute vertebral compression fracture
	42	William, C.; Paul, B.; Terry, D.; Patrick, M.; Peter, S.; Glenn, S.	2016	A controlled trial of vertebroplasty for acute painful osteoporotic fracture (vapour trial)
	43	Ranstam, J.; Turkiewicz, A.; Boonen, S.; Van Meirhaeghe, J.; Bastian, L.; Wardlaw, D.	2012	Alternative analyses for handling incomplete follow-up in the intention-to-treat analysis: the randomized controlled trial of balloon kyphoplasty versus non-surgical care for vertebral compression fracture (FREE).
	44	Weber, A.; Huysmans, S. M. D.; van Kuijk, S. M. J.; Evers, Smaa; Jutten, E. M. C.; Senden, R.; Paulus, A. T. G.; van den Bergh, J. P. W.; de Bie, R. A.; Merk, J. M. R.; Bours, S. P. G.; Hulsbosch, M.; Janssen, E. R. C.; Curfs, I.; van Hemert, W. L. W.; Schotanus, M. G. M.; de Baat, P.; Schepel, N. C.; den Boer, W. A.; Hendriks, J. G. E.; Liu, W. Y.; Kleuver, M.; Pouw, M. H.; van Hooff, M. L.; Jacobs, E.; Willems, Pcph	2022	Effectiveness and cost-effectiveness of dynamic bracing versus standard care alone in patients suffering from osteoporotic vertebral compression fractures: protocol for a multicentre, two-armed, parallel-group randomised controlled trial with 12 months of
	45	Bird, P.; Clark, W.; Diamond, T.; Schlaphoff, G.; Smerdely, P.; Gonski, P.; McNeil, P.	2016	A placebo controlled trial of vertebral fill technique vertebroplasty for acute painful osteoporotic fracture (vapour trial)
	46	Martinez, A.L.; Ullrich, B.; Schenk, P.; Schnake, K.	2022	A360: Evaluation of the OF-score and therapeutic recommendations for osteoporotic thoracolumbar fractures (EOFTT)-Monocentric results of a multicenter prospective trial
	47	Van Meirhaeghe, J.; Bastian, L.; Boonen, S.; Ranstam, J.; Tillman, J.B.; Wardlaw, D.	2012	A randomized trial of balloon kyphoplasty and non-surgical management for treating acute vertebral compression fractures: Outcomes and vertebral body kyphosis correction and surgical parameters
	48	Guo, H.; Wang, B.; Hao, D.	2015	Treatment for acute or subacute osteoporotic vertebral compression fractures: Percutaneous vertebroplasty versus facet blocking, a clinical randomized study
	49	Boucher, E.; Rosgen, B.; Lang, E.	2020	Efficacy of calcitonin for treating acute pain associated with osteoporotic vertebral compression fracture: an updated systematic review
	50	Cunningham, C.; Mc Laughlin, H.; G, O. Donoghue	2023	Physiotherapy post Vertebral Fragility Fracture: A Scoping Review
	51	Jenkinson, E.	2011	Towards evidence-based emergency medicine: best BETs from the Manchester Royal Infirmary. BET 1: do bisphosphonates relieve pain caused by acute osteoporotic vertebral compression fractures?
	52	Kan, S. L.; Yuan, Z. F.; Chen, L. X.; Sun, J. C.; Ning, G. Z.; Feng, S. Q.	2017	Which is best for osteoporotic vertebral compression fractures: balloon kyphoplasty, percutaneous vertebroplasty or non-surgical treatment? A study protocol for a Bayesian network meta-analysis

	53	L√°inez Ramos-Bossini, A.J.; L√≥pez Z√∫√±iga, D.; Ruiz Santiago, F.	2021	Percutaneous vertebroplasty versus conservative treatment and placebo in osteoporotic vertebral fractures: meta-analysis and critical review of the literature
	54	Li, K.; Gong, H.; Xie, R.; Gu, J.; Wang, S.; Lin, C.; Yin, J.; Hou, X.; Zhang, Q.; Li, L.; Hao, Y.	2021	Clinical efficacy of zoledronic acid combined with percutaneous kyphoplasty in the prevention and treatment of osteoporotic vertebral compression fracture: A systematic review and meta-analysis
	55	Li, L.; Ren, J.; Liu, J.; Wang, H.; Wang, X.; Liu, Z.; Sun, T.	2015	Results of Vertebral Augmentation Treatment for Patients of Painful Osteoporotic Vertebral Compression Fractures: A Meta-Analysis of Eight Randomized Controlled Trials
	56	Li, W.; Ou, H.; Zhang, L.; Zhang, C.; Chen, W.; Wang, H.	2022	Meta-Analysis of PKP or PVP Combined with Acupuncture in the Treatment of Osteoporotic Vertebral Compression Fractures
	57	Peckett, K. H.; Ponzano, M.; Steinke, A.; Giangregorio, L. M.	2023	Bracing and taping interventions for individuals with vertebral fragility fractures: a systematic review of randomized controlled trials with GRADE assessment
	58	Staples, M.P.; Kallmes, D.F.; Comstock, B.A.; Jarvik, J.G.; Osborne, R.H.; Heagerty, P.J.; Buchbinder, R.	2011	Effectiveness of vertebroplasty using individual patient data from two randomised placebo controlled trials: Meta-analysis
	59	Wali, A. R.; Martin, J. R.; Rennert, R.; Resnick, D. K.; Taylor, W.; Warnke, P.; Chen, C. C.	2017	Vertebroplasty for vertebral compression fractures: Placebo or effective?
	60	Xie, L.; Zhao, Z. G.; Zhang, S. J.; Hu, Y. B.	2017	Percutaneous vertebroplasty versus conservative treatment for osteoporotic vertebral compression fractures: An updated meta-analysis of prospective randomized controlled trials
**Wrong comparator**	1	Brinjikji, W.; Comstock, B. A.; Gray, L.; Kallmes, D. F.	2010	Local Anesthesia with Bupivacaine and Lidocaine for Vertebral Fracture trial (LABEL): a report of outcomes and comparison with the Investigational Vertebroplasty Efficacy and Safety Trial (INVEST)
	2	Farrokhi, M.R.; Alibai, E.; Maghami, Z.	2011	Randomized controlled trial of percutaneous vertebroplasty versus optimal medical management for the relief of pain and disability in acute osteoporotic vertebral compression fractures: Clinical article
	3	Huang, Z.; Zhang, L.	2012	Treatment of osteoporotic vertebral compressive fractures with percutaneous kyphoplasty and oral Zishengukang
	4	Jin, Y.	2023	Analysis of radiologic and clinical outcome in acute osteoporotic vertebral compression fracture: Single-agent teriparatide vs. teriparatide with subsequent vertebroplasty
	5	Tutton, S. M.; Pflugmacher, R.; Davidian, M.; Beall, D. P.; Facchini, F. R.; Garfin, S. R.	2015	KAST Study: The Kiva System As a Vertebral Augmentation Treatment-A Safety and Effectiveness Trial: A Randomized, Noninferiority Trial Comparing the Kiva System With Balloon Kyphoplasty in Treatment of Osteoporotic Vertebral Compression Fractures
	6	Werner, C.M.L.; Osterhoff, G.; Schlickeiser, J.; Jenni, R.; Wanner, G.A.; Ossendorf, C.; Simmen, H.-P.	2013	Vertebral body stenting versus kyphoplasty for the treatment of osteoporotic vertebral compression fractures: A randomized trial
	7	Yang, E. Z.; Xu, J. G.; Huang, G. Z.; Xiao, W. Z.; Liu, X. K.; Zeng, B. F.; Lian, X. F.	2016	Percutaneous Vertebroplasty Versus Conservative Treatment in Aged Patients With Acute Osteoporotic Vertebral Compression Fractures: A Prospective Randomized Controlled Clinical Study
	8	Queinnec, S.	2018	Long-term results of a single-centre pilot study at the initiative of the investigator comparing the safety and efficacy of two techniques for reduction of vertebral compression fracture
**Wrong intervention**	1	Dauri, M.; Coniglione, F.; Faria, S.; Fiori, R.; Frunzo, F.; Massari, F.; Simonetti, G.; Sabato, A.F.; Masala, S.	2009	Continuous i.v. infusion of remifentanil and intraosseous lidocaine provide better analgesia than intraosseous lidocaine alone in percutaneous vertebroplasty of osteoporotic fractures

© 2024 Alimy AR et al. *JAMA Network Open.*

	2	Deng, D.L.; Lian, Z.; Cui, W.F.; Liang, H.S.; Xiao, L.J.; Yao, G.	2019	Function of low back muscle exercise: Preventive effect of refracture analysis of postoperative vertebral fractures
	3	Frey, M.E.; DePalma, M.J.; Cifu, D.X.; Bhagia, S.M.; Carne, W.; Daitch, J.S.	2008	Percutaneous sacroplasty for osteoporotic sacral insufficiency fractures: a prospective, multicenter, observational pilot study
	4	Griffoni, C.; Lukassen, J. N. M.; Babbi, L.; Girolami, M.; Lamartina, C.; Cecchinato, R.; Gasbarrini, A.; Barbanti Brodano, G.	2020	Percutaneous vertebroplasty and balloon kyphoplasty in the treatment of osteoporotic vertebral fractures: a prospective randomized comparison
	5	Kao, F. C.; Hsu, Y. C.; Chen, T. S.; Tu, Y. K.; Liu, P. H.	2020	Effects of Injected Antiosteoporotic Medication Versus Oral Bisphosphonates on Rates of Repeated Vertebroplasty or Kyphoplasty
	6	Korovessis, P.; Repantis, T.; Vardakastanis, K.; Vitsas, V.	2014	Balloon kyphoplasty versus KIVA vertebral augmentation: Comparison of two techniques for osteoporotic vertebral body fractures: A prospective randomized study
	7	Korovessis, P.; Vardakastanis, K.; Repantis, T.; Vitsas, V.	2013	Balloon kyphoplasty versus KIVA vertebral augmentation--comparison of 2 techniques for osteoporotic vertebral body fractures: a prospective randomized study
	8	Kr√ºger, A.; Noriega, D.; Hernandez Ramajo, R.	2017	A Prospective Monocentric Randomized Pilot Study to Compare the Safety and Effectiveness of Two Vertebral Compression Fracture Reduction Techniques: 1-Year Results
	9	Liu, B.; Gan, F.; Ge, Y.; Yu, H.	2018	Clinical Efficacy Analysis of Percutaneous Kyphoplasty Combined with Zoledronic Acid in the Treatment and Prevention of Osteoporotic Vertebral Compression Fractures
	10	Noriega, D.C.; Marcia, S.; Theumann, N.; Blondel, B.; Simon, A.; Hassel, F.; Maestretti, G.; Petit, A.; Weidle, P.A.; Gonz√°lez Mandly, A.; Kaya, J.-M.; Touta, A.; Fuentes, S.; Pflugmacher, R.	2019	Safety and effectiveness of the SpineJack¬Æ system versus the KyphX Xpander¬Æ inflatable bone tamp for the reduction of vertebral compression fractures: A prospective, international, multicenter, randomized clinical trial (SAKOS study)
	11	Otten, L. A.; Bornemnn, R.; Jansen, T. R.; Kabir, K.; Pennekamp, P. H.; Wirtz, D. C.; Stuwe, B.; Pflugmacher, R.	2013	Comparison of balloon kyphoplasty with the new Kiva¬Æ VCF system for the treatment of vertebral compression fractures
	12	Park, S.Y.; Chae, I.J.; Kim, H.J.	2010	Comparative analysis of clinical outcomes in patients with osteoporotic vertebral compression fractures: Conservative treatment vs. Kyphoplasty
	13	Petersen, A.; Hartwig, E.; Koch, E.M.W.; Wollny, M.	2016	Clinical comparison of postoperative results of balloon kyphoplasty (BKP) versus radiofrequency-targeted vertebral augmentation (RF-TVA): a prospective clinical study
	14	Schwarz, F.; Lawson McLean, A.; Steinberg, A.L.; Ewald, C.; Kalff, R.; Waschke, A.	2019	Prospective randomized comparison of early versus newer-generation vertebral access devices for kyphoplasty
	15	Shen, G. W.; Wu, N. Q.; Zhang, N.; Jin, Z. S.; Xu, J.; Yin, G. Y.	2010	A prospective comparative study of kyphoplasty using the Jack vertebral dilator and balloon kyphoplasty for the treatment of osteoporotic vertebral compression fractures
	16	Vogl, T.J.; Pflugmacher, R.; Hierholzer, J.; Stender, G.; Gounis, M.; Wakhloo, A.; Fiebig, C.; Hammerstingl, R.	2013	Cement directed kyphoplasty reduces cement leakage as compared with vertebroplasty: Results of a controlled, randomized trial
	17	Wang, C.; Zhang, Y.; Chen, W.; Yan, S. L.; Guo, K. J.; Feng, S.	2021	Comparison of percutaneous curved kyphoplasty and bilateral percutaneous kyphoplasty in osteoporotic vertebral compression fractures: a randomized controlled trial
	18	Yi, H.; Chen, T.; Gan, J.; Dong, Z.; Liu, D.; Zheng, Y.; Ning, H.; Wei, Q.	2020	Effects of percutaneous kyphoplasty combined with zoledronic acid injection on osteoporotic vertebral compression fracture and bone metabolism indices
	19	Yue, Z.; Liu, R.; Yu, L.; Zhang, L.; Feng, Z.; Wang, X.	2020	Short-term Study of Bushen Jiangu Decoction Combined with PVP in the Treatment of Elderly Patients with Osteoporotic Thoracolumbar Vertebral Compression Fracture

© 2024 Alimy AR et al. *JAMA Network Open.*

	20	Lovi, A.; Teli, M.; Ortolina, A.; Costa, F.; Fornari, M.; Brayda-Bruno, M.	2009	Vertebroplasty and kyphoplasty: Complementary techniques for the treatment of painful osteoporotic vertebral compression fractures. A prospective non-randomised study on 154 patients
	21	Tutton, S.M.; Pflugmacher, R.; Davidian, M.; Beall, D.; Facchini, F.R.; Nutting Jr., C.; Hierholzer, J.; Nguyen, D.; Smith, R.; Schils, F.; Rappaport, J.; Jarzem, P.; Kallmes, D.F.; Stone, J.A.; Komlos, F.; Zucherman, J.; Kerr, E.; Alonzo, M.; Deramond, H.; Garfin, S.R.	2014	KAST study: The Kiva¬Æ system as a vertebral augmentation treatment-a safety and effectiveness trial
	22	Liu, K.; Tan, G.; Sun, W.; Lu, Q.; Tang, J.; Yu, D.	2022	Percutaneous kyphoplasty combined with zoledronic acid for the treatment of primary osteoporotic vertebral compression fracture: a prospective, multicenter study
**Wrong outcomes**	1	Andrei, D.; Popa, I.; Brad, S.; Iancu, A.; Oprea, M.; Vasilian, C.; Poenaru, D. V.	2017	The variability of vertebral body volume and pain associated with osteoporotic vertebral fractures: conservative treatment versus percutaneous transpedicular vertebroplasty
	2	Black DM; Cummings SR; Karpf DB; Cauley JA; Thompson DE; Nevitt MC; Bauer DC; Genant HK; Haskell WL; Marcus R; Ott SM; Torner JC; Quandt SA; Reiss TF; Ensrud KE; Black, D M; Cummings, S R; Karpf, D B; Cauley, J A; Thompson, D E	1996	Randomised trial of effect of alendronate on risk of fracture in women with existing vertebral fractures. Fracture Intervention Trial Research Group.
	3	Borgstr√∂m, F.; Olafsson, G.; Str√∂m, O.; Tillman, J.B.; Wardlaw, D.; Boonen, S.; Miltenburger, C.	2013	The impact of different health dimensions on overall quality of life related to kyphoplasty and non-surgical management
	4	Brinjikji, W.; Comstock, B.A.; Jarvik, J.G.; Kallmes, D.F.	2010	A detailed analysis of blinding efficacy in the investigational vertebroplasty efficacy and safety trial
	5	Chen, M.; Zhang, Y.; Zhang, L.; Wang, L.; Guo, Q.; Zhou, H.; Wang, W.; He, Y.; Xia, S.; Shao, L.	2022	The Effect of Education Intervention on Osteoporotic Fracture and Bone Mineral Density in Elderly Women With Osteoporosis: A Randomized Controlled Trial
	6	Firanescu, C. E.; de Vries, J.; Lodder, P.; Schoemaker, M. C.; Smeets, A. J.; Donga, E.; Juttmann, J. R.; Klazen, C. A. H.; Elgersma, O. E. H.; Jansen, F. H.; van der Horst, I.; Blonk, M.; Venmans, A.; Lohle, P. N. M.	2019	Percutaneous Vertebroplasty is no Risk Factor for New Vertebral Fractures and Protects Against Further Height Loss (VERTOS IV)
	7	Fritzell, P.; Ohlin, A.; Borgstr√∂m, F.	2011	Cost-effectiveness of balloon kyphoplasty versus standard medical treatment in patients with osteoporotic vertebral compression fracture: a Swedish multicenter randomized controlled trial with 2-year follow-up
	8	Giangregorio, L. M.; Gibbs, J. C.; Templeton, J. A.; Adachi, J. D.; Ashe, M. C.; Bleakney, R. R.; Cheung, A. M.; Hill, K. D.; Kendler, D. L.; Khan, A. A.; Kim, S.; McArthur, C.; Mittmann, N.; Papaioannou, A.; Prasad, S.; Scherer, S. C.; Thabane, L.; Wark, J. D.	2018	Build better bones with exercise (B3E pilot trial): results of a feasibility study of a multicenter randomized controlled trial of 12¬†months of home exercise in older women with vertebral fracture
	9	Grahn Kronhed, A. C.; Enthoven, P.; Sp√•ngeus, A.; Willerton, C.	2020	Mindfulness and Modified Medical Yoga as Intervention in Older Women with Osteoporotic Vertebral Fracture
	10	Kendler, D. L.; Marin, F.; Geusens, P.; L√≥pez-Romero, P.; Lespessailles, E.; Body, J. J.; Minisola, S.	2020	Psychotropic medications and proton pump inhibitors and the risk of fractures in the teriparatide versus risedronate VERO clinical trial
	11	Klazen, C. A.; Venmans, A.; de Vries, J.; van Rooij, W. J.; Jansen, F. H.; Blonk, M. C.; Lohle, P. N.; Juttmann, J. R.; Buskens, E.; van Everdingen, K. J.; Muller, A.; Fransen, H.; Elgersma, O. E.; Mali, W. P.; Verhaar, H. J.	2010	Percutaneous vertebroplasty is not a risk factor for new osteoporotic compression fractures: results from VERTOS II
	12	Majumdar, S. R.; McAlister, F. A.; Johnson, J. A.; Bellerose, D.; Siminoski, K.; Hanley, D. A.; Qazi, I.; Lier, D. A.; Lambert, R. G.; Russell, A. S.; Rowe, B. H.	2012	Interventions to increase osteoporosis treatment in patients with 'incidentally' detected vertebral fractures
	13	Nakamura, Toshitaka; Matsumoto, Toshio; Sugimoto, Toshitsugu; Hosoi, Takayuki; Miki, Takami; Gorai, Itsuo; Yoshikawa, Hideki; Tanaka, Yoshiya; Tanaka, Sakae; Sone, Teruki; Nakano, Tetsuo; Ito, Masako;	2014	Clinical Trials Express: fracture risk reduction with denosumab in Japanese postmenopausal women and men with osteoporosis:

© 2024 Alimy AR et al. *JAMA Network Open.*

		Matsui, Shigeyuki; Yoneda, Toshiyuki; Takami, Hideo; Watanabe, Ko; Osakabe, Taisuke; Shiraki, Masataka; Fukunaga, Masao		denosumab fracture intervention randomized placebo controlled trial (DIRECT).
	14	Resch, H.; Pietschmann, P.; Willvonseder, R.	1989	Estimated long-term effect of calcitonin treatment in acute osteoporotic spine fractures
	15	Rueda, A.L.; Andaluz, J.B.; Mart√≠nez- Ferrer, A.; Rom√°n, L.S.; Campodonico, D.; Fern√°ndez, J.M.M.; Peris, P.	2013	Risk factors for developing vertebral fractures after vertebroplasty
	16	Schnake, K. J.; Bouzakri, N.; Hahn, P.; Franck, A.; Blattert, T. R.; Zimmermann, V.; Gonschorek, O.; Ullrich, B.; Kandziora, F.; M√ºller, M.; Katscher, S.; Hartmann, F.; M√∂rk, S.; Verheyden, A.; Schinkel, C.; Piltz, S.; Olbrich, A.	2022	Multicenter evaluation of therapeutic strategies of inpatients with osteoporotic vertebral fractures in Germany
	17	Staples, M.P.; Howe, B.M.; Ringler, M.D.; Mitchell, P.; Wriedt, C.H.R.; Wark, J.D.; Ebeling, P.R.; Osborne, R.H.; Kallmes, D.F.; Buchbinder, R.	2015	New vertebral fractures after vertebroplasty: 2-year results from a randomised controlled trial
	18	Venmans, A.; Klazen, C. A.; van Rooij, W. J.; de Vries, J.; Mali, W. P.; Lohle, P. N.	2011	Postprocedural CT for perivertebral cement leakage in percutaneous vertebroplasty is not necessary--results from VERTOS II
	19	Viswanathan, V.K.; Shetty, A.P.; Sindhiya, N.; Kanna, R.M.; Rajasekaran, S.	2022	Prospective Study to Identify the Clinical and Radiologic Factors Predictive of Pseudarthrosis Development in Patients with Osteoporotic Vertebral Fractures
	20	Yi, X.; Lu, H.; Tian, F.; Wang, Y.; Li, C.; Liu, H.; Liu, X.; Li, H.	2014	Recompression in new levels after percutaneous vertebroplasty and kyphoplasty compared with conservative treatment
	21	Edidin, A. A.; Ong, K. L.; Lau, E.; Kurtz, S. M.	2011	Mortality risk for operated and nonoperated vertebral fracture patients in the medicare population
**Wrong patient population**	1	Berenson, J.; Pflugmacher, R.; Jarzem, P.; Zonder, J.; Schechtman, K.; Tillman, J. B.; Bastian, L.; Ashraf, T.; Vrionis, F.	2011	Balloon kyphoplasty versus non-surgical fracture management for treatment of painful vertebral body compression fractures in patients with cancer: a multicentre, randomised controlled trial
	2	Berenson, J.R.; Pflugmacher, R.; Jarzem, P.; Zonder, J.A.; Tillman, J.B.; Ashraf, T.; Vrionis, F.D.	2009	Final results of the first randomized trial comparing balloon kyphoplasty (BKP) to non-surgical management among cancer patients with vertebral compression fractures: marked improvement in back function, quality of life and pain in the BKP arm
	3	Boonen S; McClung MR; Eastell R; Fuleihan GE; Barton IP; Delmas P	2004	Safety and efficacy of risedronate in reducing fracture risk in osteoporotic women aged 80 and older: implications for the use of antiresorptive agents in the old and oldest old.
	4	Dai, S.; Lu, X.; Dai, N.; Shi, X.; Yang, P.; Peng, P.; Xu, F.	2022	Clinical Efficacy of Percutaneous Kyphoplasty Combined with Calcitriol and Calcium in the Treatment of Traumatic Nonosteoporotic Vertebral Compression Fractures
	5	Geusens, P.; Marin, F.; Kendler, D. L.; Russo, L. A.; Zerbini, C. A.; Minisola, S.; Body, J. J.; Lespessailles, E.; Greenspan, S. L.; Bagur, A.; Stepan, J. J.; Lakatos, P.; Casado, E.; Moericke, R.; L√≥pez-Romero, P.; Fahrleitner-Pammer, A.	2018	Effects of Teriparatide Compared with Risedronate on the Risk of Fractures in Subgroups of Postmenopausal Women with Severe Osteoporosis: The VERO Trial
	6	Gonnelli, S.; Cepollaro, C.; Pondrelli, C.; Martini, S.; Rossi, S.; Gennari, C.	1996	Ultrasound parameters in osteoporotic patients treated with salmon calcitonin: A longitudinal study
	7	M√ºller, C.W.; Pflugmacher, R.; Berenson, J.R.; Jarzem, P.; Zonder, J.; Tillman, J.B.; Ashraf, T.; Vrionis, F.D.	2010	Balloon kyphoplasty vs. Non-surgical management of vertebral compression fractures in cancer patients-A randomized controlled trial
	8	Masoudi, M.S.; Haghnegahdar, A.; Ghaffarpasand, F.; Ilami, G.	2017	Functional Recovery Following Early Kyphoplasty Versus Conservative Management in Stable Thoracuolumbar Fractures in Parachute Jumpers
	9	Olsen, C.F.; Bergland, A.	2014	The effect of exercise and education on fear of falling in elderly women with osteoporosis and a history of vertebral fracture: Results of a randomized controlled trial

	10	Osaki, M.; Okuda, R.; Saeki, Y.; Okano, T.; Tsuda, K.; Nakamura, T.; Morio, Y.; Nagashima, H.; Hagino, H.	2021	Efficiency of coordinator-based osteoporosis intervention in fragility fracture patients: a prospective randomized trial
	11	Pizones, J.; Z√∫√±iga, L.; √Ålvarez-Gonz√°lez, P.; S√°nchez-Mariscal, F.; Izquierdo, E.	2013	Two-year clinical and radiological outcomes in a prospective cohort of patients with vertebral fractures
	12	Rossini, M.; Viapiana, O.; Gatti, D.; De Terlizzi, F.; Adami, S.	2010	Capacitively coupled electric field for pain relief in patients with vertebral fractures and chronic pain
	13	Schmelzer-Schmied, N.; Cartens, C.; Meeder, P.J.; Dafonseca, K.	2009	Comparison of kyphoplasty with use of a calcium phosphate cement and non-operative therapy in patients with traumatic non-osteoporotic vertebral fractures
	14	Soreff, J.; Axdorph, G.; Bylund, P.	1982	Treatment of patients with unstable fractures of the thoracic and lumbar spine. A follow-up study of surgical and conservative treatment
	15	Wang, J.; Chen, M.; Du, J.	2016	[Therapeutic effect of conservative treatment of refracture in cemented vertebrae after percutaneous vertebroplasty for osteoporotic vertebral compression fractures]
	16	Zheng, R.-K.; Wang, Y.-S.; Li, J.-Z.; Hao, Y.-J.; Tan, H.-Y.	2013	A prospective study of percutaneous vertebroplasty for chronic painful osteoporotic spinal fractures
	17	Bastian, L.; Pfl√ºgmacher, P.R.; Berenson, J.R.; Jarzem, P.; Zonder, J.; Tillman, J.B.; Ashraf, T.; Vrionis, F.D.	2011	First randomized trial comparing balloon kyphoplasty (BKP) to non-surgical management among cancer patients with vertebral compression fractures
	18	Russo, L.A.; Lau, E.; Tang, H.; Rajman, M.; Teglbjarg, C.; Hoeck, H.C.; Alexandersen, P.; Valter, I.; Chapurlat, R.; Brandi, M.L.; Visockiene, Z.; Bone, H.G.; McClung, M.; John, M.; Loeffler, J.; Arnold, M.; Riis, B.J.; Azria, M.; Christiansen, C.	2010	Efficacy and safety of oral salmon calcitonin in postmenopausal osteoporosis: Randomized, double-blind, placebo-controlled trial
	19	Wood, K.; Butterman, G.; Mehbod, A.; Garvey, T.; Jhanjee, R.; Sechriest, V.; Buttermann, G	2003	Operative compared with nonoperative treatment of a thoracolumbar burst fracture without neurological deficit. A prospective, randomized study.
	20	Bergland, A.; Thorsen, H.; K√•resen, R.	2011	Effect of exercise on mobility, balance, and health-related quality of life In osteoporotic women with a history of vertebral fracture: A randomized, controlled trial
	21	Lyritis, G. P.; Androulakis, C.; Magiasis, B.; Charalambaki, Z.; Tsakalakos, N.	1994	Effect of nandrolone decanoate and 1-alpha-hydroxy-calciferol on patients with vertebral osteoporotic collapse. A double-blind clinical trial
	22	Chen, D.; An, Z. Q.; Song, S.; Tang, J. F.; Qin, H.	2014	Percutaneous vertebroplasty compared with conservative treatment in patients with chronic painful osteoporotic spinal fractures
	23	Kasperk, C.; Hillmeier, J.; N√∂ldge, G.; Grafe, I.A.; Dafonseca, K.; Raupp, D.; Bardenheuer, H.; Libicher, M.; Liegibel, U.M.; Sommer, U.; Hilscher, U.; Pyerin, W.; Vetter, M.; Meinzer, H.-P.; Meeder, P.-J.; Taylor, R.S.; Nawroth, P.	2005	Treatment of painful vertebral fractures by kyphoplasty in patients with primary osteoporosis: A prospective nonrandomized controlled study
	24	Medici, A.; Meccariello, L.; Falzarano, G.	2014	Non-operative vs. percutaneous stabilization in Magerl's A1 or A2 thoracolumbar spine fracture in adults: is it really advantageous for a good alignment of the spine? Preliminary data from a prospective study
	25	Bailey, C. S.; Dvorak, M. F.; Thomas, K. C.; Boyd, M. C.; Paquett, S.; Kwon, B. K.; France, J.; Gurr, K. R.; Bailey, S. I.; Fisher, C. G.	2009	Comparison of thoracolumbosacral orthosis and no orthosis for the treatment of thoracolumbar burst fractures: interim analysis of a multicenter randomized clinical equivalence trial
	26	Stadhouder, A.; Buskens, E.; Vergroesen, D. A.; Fidler, M. W.; de Nies, F.; Oner, F. C.	2009	Nonoperative treatment of thoracic and lumbar spine fractures: a prospective randomized study of different treatment options

	27	Wood, Kirkham B; Buttermann, Glenn R; Phukan, Rishabh; Harrod, Christopher C; Mehbod, Amir; Shannon, Brian; Bono, Christopher M; Harris, Mitchel B	2015	Operative compared with nonoperative treatment of a thoracolumbar burst fracture without neurological deficit: a prospective randomized study with follow-up at sixteen to twenty-two years.
**Wrong study design**	1	Bastian, L.; Van Meirhaeghe, J.; Boonen, S.; Ranstam, J.; Tillman, J.; Wardlaw, D.	2012	A randomized multicenter trial of balloon kyphoplasty and non-surgical management for treating acute vertebral compression fractures: Vertebral body kyphosis correction and quality of life
	2	Achatz, Gerhard; Riesner, Hans-Joachim; Friemert, Benedikt; Lechner, Raimund; Graf, Nicolas; Wilke, Hans-Joachim	2017	Biomechanical in vitro comparison of radiofrequency kyphoplasty and balloon kyphoplasty.
	3	Alvarez, L.; Alcaraz, M.; P√©rez-Higueras, A.; Granizo, J. J.; de Miguel, I.; Rossi, R. E.; Qui√±ones, D.	2006	Percutaneous vertebroplasty: functional improvement in patients with osteoporotic compression fractures
	4	Balkarli, H.; Kilic, M.; Balkarli, A.; Erdogan, M.	2016	An evaluation of the functional and radiological results of percutaneous vertebroplasty versus conservative treatment for acute symptomatic osteoporotic spinal fractures
	5	Bergland, A.	2012	Effect of exercise on falls efficacy in osteoporotic women with a history of vertebral fracture: A randomized, controlled trial
	6	Bertoldo, F.; Gandolini, G.; Venturin, A.; Cisari, C.; Lovato, R.; Longhi, M.; Farina, S.; Bertoldo, E.; Nuti, R.	2018	Pain relief management of acute osteoporotic vertebral fracture in a real life study
	7	Blasco, J.; Martinez-Ferrer, A.; MacHo, J.; San Roman, L.; Pom√©s, J.; Carrasco, J.; Monegal, A.; Gua√±abens, N.; Peris, P.	2012	Effect of vertebroplasty on pain relief, quality of life, and the incidence of new vertebral fractures: A 12-month randomized follow-up, controlled trial
	8	Boonen, S.; Van Meirhaeghe, J.; Bastian, L.; Cummings, S. R.; Ranstam, J.; Tillman, J. B.; Eastell, R.; Talmadge, K.; Wardlaw, D.	2011	Balloon kyphoplasty for the treatment of acute vertebral compression fractures: 2-year results from a randomized trial
	9	Bornemann, R.; Hanna, M.; Kabir, K.; Goost, H.; Wirtz, D. C.; Pflugmacher, R.	2012	Continuing conservative care versus crossover to radiofrequency kyphoplasty: a comparative effectiveness study on the treatment of vertebral body fractures
	10	Brinjikji, W.; Comstock, B.A.; Heagerty, P.J.; Jarvik, J.G.; Kallmes, D.F.	2010	Investigational vertebroplasty efficacy and safety trial: Detailed analysis of blinding efficacy
	11	Brinjikji, W.; Comstock, B.A.; Jarvik, J.G.; Kallmes, D.F.	2010	Did patients with severe pain improve in INVEST?
	12	Buchbinder, R.; Osborne, R.H.; Ebeling, P.R.; Wark, J.D.; Mitchell, P.; Wriedt, C.; Graves, S.; Staples, M.P.; Murphy, B.	2009	A randomized trial of vertebroplasty for painful osteoporotic vertebral fractures
	13	Cai, P.; Zhou, C. X.; Li, D.; Chen, Y. L.; Chang, Z. Y.; Xie, L.	2020	[Analgesic effect of combined therapy of medication and acupuncture in PVP for osteoporotic vertebral compression fracture: a randomized controlled trial]
	14	Chabert, E.; Hugonnet, E.; Kastler, A.; Sakka, L.; Rabbo, F. A.; Zerroug, A.; Coudeyre, E.; Pereira, B.; Coll, G.	2023	Vertebroplasty versus bracing in acute vertebral compression fractures: A prospective randomized trial
	15	Chen, B.L.; Zhong, Y.; Huang, Y.L.; Zeng, L.W.; Li, Y.Q.; Yang, X.X.; Jiang, Q.; Wang, C.H.	2012	Systematic back muscle exercise after percutaneous vertebroplasty for spinal osteoporotic compression fracture patients: a randomized controlled trial.
	16	Chen, J.-P.; Qi, X.-W.; Li, S.-J.; Kuang, L.-P.; Yuan, X.-H.; Wang, G.-S.; Tan, W.-Y.	2015	Bone cement injection as vertebral augmentation therapy for osteoporotic vertebral compression fractures
	17	Chen, X. M.; Ma, H. S.; Wang, M.; Yang, B.; Yuan, W.	2013	Analysis of causes of pain after kyphoplasty for osteoporotic vertebral compression fractures
	18	Chosa, K.; Naito, A.; Awai, K.	2011	Newly developed compression fractures after percutaneous vertebroplasty: comparison with conservative treatment
	19	Clark, W.; Bird, P.; Gonski, P.; Diamond, T.H.; Smerdely, P.; McNeil, H.P.; Schlaphoff, G.; Bryant, C.; Barnes, E.; Gebski, V.	2016	Safety and efficacy of vertebroplasty for acute painful osteoporotic fractures (VAPOUR): a multicentre, randomised, double-blind, placebo-controlled trial

© 2024 Alimy AR et al. *JAMA Network Open.*

	20	Comstock, B. A.; Sitlani, C. M.; Jarvik, J. G.; Heagerty, P. J.; Turner, J. A.; Kallmes, D. F.	2013	Investigational vertebroplasty safety and efficacy trial (INVEST): patient-reported outcomes through 1 year
	21	Dave, M.; Dave, B.; Krishnan, A.; Deglumadi, D.; Mayi, S.; Rai, R.; Dave, A.	2022	A208: Teriparatide (TPTD) as the first line of treatment for acute, uncomplicated osteoporotic vertebral compression fractures (OVCF), with percutaneous vertebroplasty (PVP) reserved for failed and non-union patient group-A prospective observational study
	22	Diamond, T. H.; Champion, B.; Clark, W. A.	2003	Management of acute osteoporotic vertebral fractures: a nonrandomized trial comparing percutaneous vertebroplasty with conservative therapy
	23	Diamond, T.; Clark, W.; Bird, P.; Gonski, P.; Barnes, E.; Gebski, V.	2020	Early vertebroplasty within 3¬†weeks of fracture for acute painful vertebral osteoporotic fractures: subgroup analysis of the VAPOUR trial and review of the literature
	24	Diamond, T.H.; Bryant, C.; Browne, L.; Clark, W.A.	2006	Clinical outcomes after acute osteoporotic vertebral fractures: A 2-year non-randomised trial comparing percutaneous vertebroplasty with conservative therapy
	25	Du, J.P.; Fan, Y.; Liu, J.J.; Zhang, J.N.; Huang, Y.S.; Zhang, J.; Hao, D.J.	2018	The analysis of MSTMOVCF (Multi-segment thoracolumbar mild osteoporotic fractures surgery or conservative treatment) based on ASTLOF (the assessment system of thoracolumbar osteoporotic fracture)
	26	Dudeney, S.; Lieberman, I.	2000	Percutaneous vertebroplasty in the treatment of osteoporotic vertebral compression fractures: an open prospective study
	27	Eidt-Koch, D.; Greiner, W.	2011	Quality of life results of balloon kyphoplasty versus non surgical management for osteoporotic vertebral fractures in Germany
	28	Faloon, M. J.; Ruoff, M.; Deshpande, C.; Hohman, D.; Dunn, C.; Beckloff, N.; Patel, D. V.	2015	Risk Factors Associated with Adjacent and Remote- Level Pathologic Vertebral Compression Fracture Following Balloon Kyphoplasty: 2-Year Follow-Up Comparison Versus Conservative Treatment
	29	Ferdinandov, D.; Yankov, D.	2020	Features and algorithm of surgical behavior in osteoporotic vertebral fractures
	30	Firanescu, C. E.; de Vries, J.; Lodder, P.; Venmans, A.; Schoemaker, M. C.; Smeets, A. J.; Donga, E.; Juttmann, J. R.; Klazen, C. A. H.; Elgersma, O. E. H.; Jansen, F. H.; Tielbeek, A. V.; Boukrab, I.; Schonenberg, K.; van Rooij, W. J. J.; Hirsch, J. A.; Lohle, P. N. M.	2018	Vertebroplasty versus sham procedure for painful acute osteoporotic vertebral compression fractures (VERTOS IV): randomised sham controlled clinical trial
	31	Firanescu, C. E.; Venmans, A.; de Vries, J.; Lodder, P.; Schoemaker, M. C.; Smeets, A. J.; Donga, E.; Juttmann, J. R.; Schonenberg, K.; Klazen, C. A. H.; Elgersma, O. E. H.; Jansen, F. H.; Fransen, H.; Hirsch, J. A.; Lohle, P. N. M.	2022	Predictive Factors for Sustained Pain after (sub)acute Osteoporotic Vertebral Fractures. Combined Results from the VERTOS II and VERTOS IV Trial
	32	Firanescu, C.E.	2013	VERTOS IV trial: A randomised controlled trial of vertebroplasty for painful acute osteoporotic vertebral fractures using a sham procedure as control
	33	Giannotti, S.; Carmassi, F.; Bottai, V.; Dell'osso, G.; Gazzarri, F.; Guido, G.	2012	Comparison of 50 vertebral compression fractures treated with surgical (kyphoplasty) or non surgical approach
	34	Grafe, I.A.; Da Fonseca, K.; Hillmeier, J.; Meeder, P.-J.; Libicher, M.; N√∂ldge, G.; Bardenheuer, H.; Pyerin, W.; Basler, L.; Weiss, C.; Taylor, R.S.; Nawroth, P.; Kasperk, C.	2005	Reduction of pain and fracture incidence after kyphoplasty: 1-Year outcomes of a prospective controlled trial of patients with primary osteoporosis
	35	Grey A; Bolland M; Wong S; Horne A; Gamble G; Reid IR	2012	Low-dose zoledronate in osteopenic postmenopausal women: a randomized controlled trial.
	36	Guo, H.; Hao, D.; Wang, B.	2016	Treatment of pain due to osteoporotic vertebral compression fractures: A randomized prospective study of percutaneous vertebroplasty versus facet blocking

	37	Hao, D.; Wang, B.; Guo, H.; Wang, X.	2014	Treatment for acute or subacute osteoporosis vertebral compression fractures: Percutaneous vertebroplasty versus facets blocking (a clinical randomized study)
	38	Hao, D.J.; Xie, E.; Wu, Q.N.	2013	Percutaneous kyphoplasty versus conservative treatment in acute and subacute osteoporotic vertebral compression fractures (OVCF): A double-blinded, randomized controlled clinical trial (RCT) in the population of western China
	39	Hoshino, M.; Takahashi, S.; Yasuda, H.; Terai, H.; Watanabe, K.; Hayashi, K.; Tsujio, T.; Kono, H.; Suzuki, A.; Tamai, K.; Ohyama, S.; Toyoda, H.; Dohzono, S.; Kanematsu, F.; Hori, Y.; Nakamura, H.	2019	Balloon Kyphoplasty Versus Conservative Treatment for Acute Osteoporotic Vertebral Fractures with Poor Prognostic Factors: Propensity Score Matched Analysis Using Data from Two Prospective Multicenter Studies
	40	Hou, X. Y.; Xu, S. H.; Dai, Z. Y.; Yang, J. X.	2021	[Analysis on application value of Fu's subcutaneous needling for intercostal pain after surgery of osteoporotic thoracic vertebral compression fracture]
	41	Ishiguro, S.; Kasai, Y.; Sudo, A.; Iida, K.; Uchida, A.	2010	Percutaneous vertebroplasty for osteoporotic compression fractures using calcium phosphate cement
	42	Isik, N.; Ullrich, B.W.; Katscher, S.; Schenk, P.; Schnake, K.J.; Spiegl, U.J.; Schmeiser, G.; Zimmermann, V.; Perl, M.; Scherer, M.A.; Jacobi, A.	2019	Clinical treatment outcomes and complications of osteoporotic thoracolumbar fractures after conservative or surgical treatment. Initial data from the prospective, multicenter study evaluating the of classification, OF-score and therapy recommendation for
	43	Jarzem, P.; Pflugmacher, R.; Berenson, J.; Zonder, J.; Tillman, J.; Bastian, L.; Ashraf, T.; Vrionis, F.	2011	Balloon kyphoplasty improves quality of life, bodily pain and vertebral body height among cancer patients with vertebral compression fractures compared to nonsurgical management: Results from a multicenter, randomized trial
	44	Jin, C.; Xu, G.; Weng, D.; Xie, M.; Qian, Y.	2018	Impact of magnetic resonance imaging on treatment-related decision making for osteoporotic vertebral compression fracture: A prospective randomized trial
	45	Kallmes, D. F.; Comstock, B. A.; Heagerty, P. J.; Turner, J. A.; Wilson, D. J.; Diamond, T. H.; Edwards, R.; Gray, L. A.; Stout, L.; Owen, S.; Hollingworth, W.; Ghdoke, B.; Annesley-Williams, D. J.; Ralston, S. H.; Jarvik, J. G.	2009	A randomized trial of vertebroplasty for osteoporotic spinal fractures
	46	Kallmes, D.F.; Comstock, B.A.; Gray, L.A.; Heagerty, P.J.; Hollingworth, W.; Turner, J.A.; Stout, L.; Jarvik, J.G.	2009	Baseline pain and disability in the investigational vertebroplasty efficacy and safety trial
	47	Kaneb, A.; Berardino, K.; Hanukaai, J. S.; Rooney, K.; Kaye, A. D.	2021	Calcitonin (FORTICAL, MIACALCIN) for the treatment of vertebral compression fractures
	48	Kasperk, C.; Grafe, I.A.; Schmitt, S.; N√∂ldge, G.; Weiss, C.; Da Fonseca, K.; Hillmeier, J.; Libicher, M.; Sommer, U.; Rudofsky, G.; Meeder, P.-J.; Nawroth, P.	2010	Three-year outcomes after kyphoplasty in patients with Osteoporosis with painful vertebral fractures
	49	Khoury, N.; Batista, A.L.; Cloutier, F.; Weill, A.; Roy, D.; Raymond, J.	2016	Vertebroplasty in the treatment of acute fracture (VITTA) trial
	50	Kim, Y. C.; Bok, D. H.; Chang, H. G.; Kim, S. W.; Park, M. S.; Oh, J. K.; Kim, J.; Kim, T. H.	2016	Increased sagittal vertical axis is associated with less effective control of acute pain following vertebroplasty
	51	Klazen, C. A.; Lohle, P. N.; de Vries, J.; Jansen, F. H.; Tielbeek, A. V.; Blonk, M. C.; Venmans, A.; van Rooij, W. J.; Schoemaker, M. C.; Juttmann, J. R.; Lo, T. H.; Verhaar, H. J.; van der Graaf, Y.; van Everdingen, K. J.; Muller, A. F.; Elgersma, O. E.; Halkema, D. R.; Fransen, H.; Janssens, X.; Buskens, E.; Mali, W. P.	2010	Vertebroplasty versus conservative treatment in acute osteoporotic vertebral compression fractures (Vertos II): an open-label randomised trial

© 2024 Alimy AR et al. *JAMA Network Open.*

	52	Klazen, C. A.; Verhaar, H. J.; Lohle, P. N.; Lampmann, L. E.; Juttmann, J. R.; Schoemaker, M. C.; van Everdingen, K. J.; Muller, A. F.; Mali, W. P.; de Vries, J.	2010	Clinical course of pain in acute osteoporotic vertebral compression fractures
	53	Klazen, C.A.; Lohle, P.N.; De Vries, J.; Jansen, F.H.; Tielbeek, A.V.; Venmans, A.; Schoemaker, M.C.; Lo, T.H.; Fransen, H.; Elgersma, O.E.; Van Everdingen, K.J.; Buskens, E.; Verhaar, H.J.; Mali, W.P.	2010	Percutaneous vertebroplasty versus conservative therapy in patients with an acute osteoporotic vertebral compression fracture. Vertos II: A randomized controlled trial
	54	Klezl, Z.; Bhangoo, N.; Phillips, J.; Swamy, G.; Calthorpe, D.; Bommireddy, R.	2012	Social implications of balloon kyphoplasty: Prospective study from a single UK centre
	55	Kroon, F.; Staples, M.; Ebeling, P.R.; Wark, J.D.; Osborne, R.H.; Mitchell, P.J.; Wriedt, C.H.R.; Buchbinder, R.	2014	Two-year results of a randomized placebo-controlled trial of vertebroplasty for acute osteoporotic vertebral fractures
	56	Kuntz, D.; Marie, P.; Berhel, M.; Caulin, F.	1986	Treatment of post-menopausal osteoporosis with phosphate and intermittent calcitonin
	57	Leali, P. T.; Solla, F.; Maestretti, G.; Balsano, M.; Doria, C.	2016	Safety and efficacy of vertebroplasty in the treatment of osteoporotic vertebral compression fractures: a prospective multicenter international randomized controlled study
	58	Lee, H. M.; Park, S. Y.; Lee, S. H.; Suh, S. W.; Hong, J. Y.	2012	Comparative analysis of clinical outcomes in patients with osteoporotic vertebral compression fractures (OVCFs): conservative treatment versus balloon kyphoplasty
	59	Lin, C.; Yu, C.; Chen, L.; Ke, Z.; Deng, Z.	2011	[Comparison of effectiveness between kyphoplasty and conservative treatment in treating osteoporotic vertebral fractures].
	60	Lin, F.; Zhang, Y.; Wu, T.; Niu, Y.; Su, P.; Hua, J.; Sun, Y.	2022	Local Anesthetic and Steroid Injection to Relieve the Distal Lumbosacral Pain in Osteoporotic Vertebral Compression Fractures of Patients Treated with Kyphoplasty
	61	Liu, T.; Qiu, S.; Xu, Z.; Gu, J.; Luo, Z.; Wu, D.	2019	[Effect of percutaneous kyphoplasty on lumbar-pelvic correlation in osteoporotic vertebral compressive fractures]
	62	Liu, W.; Gao, G.; L√º, J.; Sun, J.; Wang, Y.; Li, Z.; Wang, H.	2015	[Vertebroplasty compared with conservative method of integrated Chinese and Western Medicine in patients with osteoporotic vertebral compression fractures]
	63	Longo, U. G.; Loppini, M.; Denaro, L.; Brandi, M. L.; Maffulli, N.; Denaro, V.	2010	The effectiveness and safety of vertebroplasty for osteoporotic vertebral compression fractures. A double blind, prospective, randomized, controlled study
	64	Lu, K.; Yin, Y.; Li, C.; Jin, Y.; Shan, H. Q.	2021	Efficacy of annual zoledronic acid in initial percutaneous kyphoplasty patients with osteoporotic vertebral compression fractures: a 3-year follow-up study
	65	M√ºller, C. W.; G√∂sling, T.; Mameghani, A.; Stier, R.; Klein, M.; H√ºfner, T.; Krettek, C.	2010	[Vertebral fractures due to osteoporosis. Kyphoplasty and vertebroplasty vs conservative treatment]
	66	Ma, Y.; Wu, X.; Xiao, X.; Feng, L.; Yan, W.; Chen, J.; Yang, D.	2020	Effects of teriparatide versus percutaneous vertebroplasty on pain relief, quality of life and cost-effectiveness in postmenopausal females with acute osteoporotic vertebral compression fracture: A prospective cohort study
	67	Mac√≠as-Hern√°ndez, S.I.; Ch√°vez-Arias, D.D.; Miranda-Duarte, A.; Coronado-Zarco, R.; Diez-Garc√≠a, M.P.	2015	Percutaneous Vertebroplasty Versus Conservative Treatment and Rehabilitation in Women with Vertebral Fractures due to Osteoporosis: A Prospective Comparative Study
	68	Manzini, C.U.; Bernini, L.; Vallone, S.; Cavalleri, F.; Ferri, C.	2007	Percutaneous vertebroplasty as therapy of vertebral fractures: Results in a series of osteoporotic patients
	69	Martinez-Ferrer, √Ä.; Blasco, J.; Carrasco, J.L.; Monegal, A.; Gua√±abens, N.; Peris, P.	2012	Effect of vertebroplasty on pain relief, quality of life and in the incidence of new vertebral fractures. A 12-month randomized follow-up, controlled trial

	70	Martinez, A.L.; Schenk, P.; Schnake, K.J.; Ullrich, B.W.	2022	Evaluation of the OF-score and therapeutic recommendations for osteoporotic thoracolumbar fractures (EOFTT) - monocentric results of a multicenter prospective trial
	71	Matsumoto T; Hagino H; Shiraki M; Fukunaga M; Nakano T; Takaoka K; Morii H; Ohashi Y; Nakamura T	2009	Effect of daily oral minodronate on vertebral fractures in Japanese postmenopausal women with established osteoporosis: a randomized placebo-controlled double-blind study.
	72	McDonald, R. J.; Achenbach, S. J.; Atkinson, E. J.; Gray, L. A.; Cloft, H. J.; Melton, L. J., 3rd; Kallmes, D. F.	2011	Mortality in the vertebroplasty population
	73	Moretti, A.; de Sire, A.; Curci, C.; Toro, G.; Gimigliano, F.; Iolascon, G.	2019	Effectiveness of denosumab on back pain-related disability and quality-of-life in patients with vertebral fragility fractures
	74	Moretti, A.; Gimigliano, F.; Di Pietro, G.; Gimigliano, R.; Iolascon, G.	2015	Back pain-related disability and quality of life in patients affected by vertebral fractures: data from baseline characteristics of population enrolled in Denosumab In Real Practice (DIRP)
	75	Movrin, I.	2012	Adjacent level fracture after osteoporotic vertebral compression fracture: A nonrandomized prospective study comparing balloon kyphoplasty with conservative therapy
	76	Nakamura, T.; Fukunaga, M.; Nakano, T.; Kishimoto, H.; Ito, M.; Hagino, H.; Sone, T.; Taguchi, A.; Tanaka, S.; Ohashi, M.; Ota, Y.; Shiraki, M.	2017	Efficacy and safety of once-yearly zoledronic acid in Japanese patients with primary osteoporosis: two-year results from a randomized placebo-controlled double-blind study (ZOledroNate treatment in Efficacy to osteoporosis; ZONE study).
	77	Noriega, D.C.	2019	Long-term safety and clinical performance of kyphoplasty and SpineJack procedures in the treatment of osteoporotic vertebral compression fractures: A pilot, monocentric
	78	Oh, Y.; Lee, B.; Lee, S.; Kim, J.; Park, J.	2019	Percutaneous Vertebroplasty versus Conservative Treatment Using a Transdermal Fentanyl Patch for Osteoporotic Vertebral Compression Fractures
	79	Peris, P.; Blasco, J.; Carrasco, J.L.; Martinez-Ferrer, A.; Macho, J.; Rom√°n, L.S.; Monegal, A.; Gua√±abens, N.	2015	Risk Factors for the Development of Chronic Back Pain After Percutaneous Vertebroplasty Versus Conservative Treatment
	80	Qian, H.; Zhou, J.; Huang, T.; Cao, X.; Zhou, C.; Yang, M.; Chen, Y.	2021	Comfort nursing can alleviate pain and negative emotion of patients after surgery for LVCFs and improve their living ability
	81	Qian, J.; Yang, H.; Jing, J.; Zhao, H.; Ni, L.; Tian, D.; Wang, Z.	2012	The early stage adjacent disc degeneration after percutaneous vertebroplasty and kyphoplasty in the treatment of osteoporotic VCFs
	82	Rousing, R.; Andersen, M. O.; Jespersen, S. M.; Thomsen, K.; Lauritsen, J.	2009	Percutaneous vertebroplasty compared to conservative treatment in patients with painful acute or subacute osteoporotic vertebral fractures: three-months follow-up in a clinical randomized study
	83	Rousing, R.; Hansen, K.L.; Andersen, M.O.; Jespersen, S.M.; Thomsen, K.; Lauritsen, J.M.	2010	Twelve-months follow-up in forty-nine patients with acute/semiacute osteoporotic vertebral fractures treated conservatively or with percutaneous vertebroplasty: A clinical randomized study
	84	Shu, L. J.; Zhang, J. Y.	2022	Effect of Artificial Tiger Bone Powder (Jintiange Capsule) on Vertebral Height Ratio, Cobb's Angle, Bone Mineral Density, and Visual Analog Score
	85	Smith, F.W.; Boonen, S.; Van Meirhaeghe, J.; Bastian, L.; Wardlaw, D.	2009	A randomized trial of balloon kyphoplasty and nonsurgical care for acute vertebral compression fracture: 2-year results
	86	Sontag A; Wan X; Krege JH	2010	Benefits and risks of raloxifene by vertebral fracture status.
	87	Spiegl, U. J. A.; Schenk, P.; Schnake, K. J.; Ullrich, B. W.; Osterhoff, G.; Scheyerer, M. J.; Schmeiser, G.; B√§umlein, M.; Scherer, M. A.; M√ºller,	2023	Treatment and Outcome of Osteoporotic Thoracolumbar Vertebral Body Fractures With Deformation of Both Endplates With or Without

© 2024 Alimy AR et al. *JAMA Network Open.*

		M.; Sprengel, K.; Liepold, K.; Schramm, S.; Baron, H. C.; Siekmann, H.; Schwarz, F.; Franck, A.; Zimmermann, V.; Katscher, S.		Posterior Wall Involvement (OF 4): Short-Term Results from the Prospective EOFTT Multicenter Study
	88	Sup, K.; Yang, J.J.	2011	Recollapse of osteoporotic vertebral fractures: Nonrandomized trial comparing percutaneous vertebroplasty with conservative treatment song
	89	Tan, C. W.; Cameron, M.; Arlachov, Y.; Bastounis, A.; Bishop, S.; Czernicki, M.; Drummond, A.; Fakis, A.; Pasku, D.; Sahota, O.	2022	The Acute VertEbRal AugmentaTion (AVERT) study: protocol for a randomised controlled, feasibility trial of spinal medial branch nerve block in hospitalised older patients with vertebral fragility fractures
	90	Tang, C.; Tang, X.; Zhang, W.; Dai, M.; Peng, M.; He, S.	2021	Percutaneous mesh-container-plasty for osteoporotic thoracolumbar burst fractures: A prospective, nonrandomized comparative study
	91	Tantawy, M.F.	2022	Efficacy and safety of percutaneous vertebroplasty for osteoporotic vertebral compression fractures
	92	Thillainadesan, J.; Schlaphoff, G.; Gibson, K. A.; Hassett, G. M.; McNeil, H. P.	2010	Long-term outcomes of vertebroplasty for osteoporotic compression fractures
	93	Tseng, Y. Y.; Su, C. H.; Lui, T. N.; Yeh, Y. S.; Yeh, S. H.	2012	Prospective comparison of the therapeutic effect of teriparatide with that of combined vertebroplasty with antiresorptive agents for the treatment of new-onset adjacent vertebral compression fracture after percutaneous vertebroplasty
	94	Ullrich, B. W.; Schnake, K. J.; Schenk, P.; Katscher, S.; B√§umlein, M.; Zimmermann, V.; Schwarz, F.; Schmeiser, G.; Scherer, M.; M√ºller, M.; Sprengel, K.; Liepold, K.; Schramm, S.; Baron, H. C.; Siekmann, H.; Franck, A.; Scheyerer, M. J.; Kirtas, S.; Spiegl, U. J. A.; Osterhoff, G.	2023	Clinical Evaluation of the Osteoporotic Fracture Treatment Score (OF-Score): Results of the Evaluation of the Osteoporotic Fracture Classification, Treatment Score and Therapy Recommendations (EOFTT) Study
	95	Ullrich, B.; Katscher, S.; Schnake, K.J.; Baumlein, M.; Zimmermann, V.; Schwarz, F.; Schmeiser, G.; Scherer, M.; Muller, M.; Sprengel, K.; Spiegl, U.; Liepold, K.; Baron, H.C.; Perl, M.; Siekmann, H.; Piltz, S.; Scheyerer, M.J.; Sektion Wirbelsaule, A.O.	2021	Inpatient care reality and complications of treatment of acute osteoporotic fractures of the thoracolumbar spine-Results of the EOFTT Study
	96	Van Meirhaeghe, J.; Bastian, L.; Boonen, S.; Ranstam, J.; Tillman, J. B.; Wardlaw, D.	2013	A randomized trial of balloon kyphoplasty and nonsurgical management for treating acute vertebral compression fractures: vertebral body kyphosis correction and surgical parameters
	97	Van Meirhaeghe, J.K.; Bastian, L.; Boonen, S.; Ranstam, J.; Wardlaw, D.	2012	A randomized trial of balloon kyphoplasty and non-surgical management for treating acute vertebral compression fractures: Vertebral body kyphosis correction and surgical parameters
	98	Venmans, A.; Klazen, C. A.; Lohle, P. N.; Mali, W. P.; van Rooij, W. J.	2012	Natural history of pain in patients with conservatively treated osteoporotic vertebral compression fractures: results from VERTOS II
	99	Venmans, A.; Klazen, C.A.H.; Lohle, P.N.M.; Van Rooij, W.J.; Verhaar, H.J.J.; De Vries, J.; Mali, W.P.Th.M.	2010	Percutaneous vertebroplasty and pulmonary cement embolism: Results from VERTOS II
	100	Venmans, A.; Lohle, P. N.; van Rooij, W. J.	2014	Pain course in conservatively treated patients with back pain and a VCF on the spine radiograph (VERTOS III)
	101	Voormolen, M. H.; Mali, W. P.; Lohle, P. N.; Fransen, H.; Lampmann, L. E.; van der Graaf, Y.; Juttmann, J. R.; Jansssens, X.; Verhaar, H. J.	2007	Percutaneous vertebroplasty compared with optimal pain medication treatment: short-term clinical outcome of patients with subacute or chronic painful osteoporotic vertebral compression fractures. The VERTOS study
	102	Wang Lihong; Yu Haiqian	2017	Nursing care of 24 patients of osteoporotic vertebral compression fracture treated with kyphoplasty.
	103	Wang, B.; Guo, H.; Yuan, L.; Huang, D.; Zhang, H.; Hao, D.	2016	A prospective randomized controlled study comparing the pain relief in patients with osteoporotic vertebral compression fractures with the use of vertebroplasty or facet blocking

© 2024 Alimy AR et al. *JAMA Network Open.*

	104	Wang, D.; Cang, D.; Wu, Y.; Wang, S.	2020	Therapeutic effect of percutaneous vertebroplasty and nonoperative treatment on osteoporotic vertebral compression fracture: A randomized controlled trial protocol
	105	Wang, H. K.; Lu, K.; Liang, C. L.; Weng, H. C.; Wang, K. W.; Tsai, Y. D.; Hsieh, C. H.; Liliang, P. C.	2010	Comparing clinical outcomes following percutaneous vertebroplasty with conservative therapy for acute osteoporotic vertebral compression fractures
	106	Wang, Q.; Wang, S.; Li, X.; Wei, Y.; Ma, X.	2018	Clinical observation of percutaneous vertebroplasty in the treatment of senile osteoporosis complicated with vertebral compression fractures
	107	Wang, X.; Xu, B.; Ye, X.; Yang, Y.; Wang, G.	2015	Effects of different treatments on patients with osteoporotic fracture after percutaneous kyphoplasty
	108	Wardlaw, D.; Boonen, S.; Tillman, J.	2012	A randomized trial of balloon kyphoplasty and nonsurgical management for treating acute vertebral compression fractures: Outcomes and vertebral body kyphosis correction and surgical parameters
	109	Wardlaw, D.; Cummings, S.R.; Van Meirhaeghe, J.; Bastian, L.; Tillman, J.B.; Ranstam, J.; Eastell, R.; Shabe, P.; Talmadge, K.; Boonen, S.	2009	Efficacy and safety of balloon kyphoplasty compared with non-surgical care for vertebral compression fracture (FREE): a randomised controlled trial
	110	Yakhelef, N.; Audibert, M.; Peirera, B.; Mons, A.	2016	Cost-utility analysis of vertebroplasty versus thoracolumbosacral orthosis in the treatment of traumatic vertebral fractures
	111	Yang, W.; Song, J.; Liang, M.; Cui, H.; Chen, H.; Yang, J.	2019	Functional Outcomes and New Vertebral Fractures in Percutaneous Vertebroplasty and Conservative Treatment of Acute Symptomatic Osteoporotic Vertebral Compression Fractures
	112	Yu, D.; Liu, Z.; Wang, H.; Yao, R.; Li, F.; Yang, Y.; Sun, F.	2022	Treatment of Elderly Patients with Acute Symptomatic OVCF: A Study of Comparison of Conservative Treatment and Percutaneous Kyphoplasty
	113	Yu, L.; Wen, J.; Song, H.; Liu, H.	2015	Application of Jintian'ge capsule in treatment of vertebral compression fracture in senile osteoporosis patients
	114	Kong, M.; Zhou, C.; Zhu, K.; Zhang, Y.; Song, M.; Zhang, H.; Tu, Q.; Ma, X.	2019	12-Month Teriparatide Treatment Reduces New Vertebral Compression Fractures Incidence And Back Pain And Improves Quality Of Life After Percutaneous Kyphoplasty In Osteoporotic Women
	115	Ravikumar, T.V.; Subithchan, P.; Mahesh, M.; Reddy, D.; Jain, V.	2022	Comparative Analysis of Conservative versus Cement Augmentation in Osteoporotic Vertebral Fractures , A Prospective and Retrospective Study
	116	Singh, V.; Taunk, A.; Phadke, R.V.; Neyaz, Z.; Prasad, S.N.	2019	Analysis of percutaneous vertebroplasty,a prospective study
	117	Klezl, Z.; Bhangoo, N.; Leung, Y.; Calthorpe, D.; Bommireddy, R.	2010	Social drift and mortality in patients treated conservatively and with kyphoplasty: prospective independent single center UK study
	118	Van Meirhaeghe, J.K.; Boonen, S.; Bastian, L.; Cummings, S.; Ranstam, J.; Tillman, J.; Eastell, R.; Talmadge, K.; Wardlaw, D.	2010	A randomized trial of balloon kyphoplasty and nonsurgical care for patients with acute vertebral compression fractures: Two year results
	119	En, X.; Hao, D.-J.	2013	Percutaneous kyphoplasty versus conservative treatment in acute and subacute osteoporotic vertebral compression fractures (OVCF): A double-blinded, randomized controlled clinical trial (RCT) in the population of Western China
	120	Umehara, T.; Inukai, A.; Kuwahara, D.; Kaneyashiki, R.; Kaneguchi, A.; Tsunematsu, M.; Kakehashi, M.	2022	Physical Functions and Comorbidity Affecting Collapse at 4 or More Weeks after Admission in Patients with Osteoporotic Vertebral Fractures: A Prospective Cohort Study
	121	Klazen, C. A.; Verhaar, H. J.; Lampmann, L. E.; Juttmann, J. R.; Blonk, M. C.; Jansen, F. H.; Tielbeek, A. V.; Schoemaker, M. C.; Buskens, E.;	2007	VERTOS II: percutaneous vertebroplasty versus conservative therapy in patients with painful osteoporotic vertebral compression fractures;

© 2024 Alimy AR et al. *JAMA Network Open.*

		van der Graaf, Y.; Janssens, X.; Fransen, H.; van Everdingen, K. J.; Muller, A. F.; Mali, W. P.; Lohle, P. N.		rationale, objectives and design of a multicenter randomized controlled trial
	122	G√∞l, S.M.; Ullrich, B.W.; Spiegl, U.J.; Schenk, P.; Schnake, K.J.; Katscher, S.; Schmeiser, G.; Zimmermann, V.; Perl, M.; Scherer, M.A.; Jacobi, A.; Isik, N.	2019	Surgical and conservative treatment of osteoporotic vertebral fractures in terms of OF-score and therapy recommendation. Initial data from the prospective, multi-center study evaluating of classification, OF-score and therapy recommendation for osteoporot
	123	D'Oria, S.; Dibenedetto, M.; Squillante, E.; Somma, C.; Hannan, C. J.; Giraldi, D.; Fanelli, V.	2022	Traumatic compression fractures in thoracic-lumbar junction: vertebroplasty vs conservative management in a prospective controlled trial
	124	Choi, J. H.; Kang, H. D.; Park, J. H.; Gu, B. S.; Jung, S. K.; Oh, S. H.	2017	The Efficacy of Fentanyl Transdermal Patch as the First-Line Medicine for the Conservative Treatment of Osteoporotic Compression Fracture
	125	Tu, P. H.; Liu, Z. H.; Lee, S. T.; Chen, J. F.	2012	Treatment of repeated and multiple new-onset osteoporotic vertebral compression fractures with teriparatide
	126	Jacobs, E.; Senden, R.; McCrum, C.; van Rhijn, L. W.; Meijer, K.; Willems, P. C.	2019	Effect of a semirigid thoracolumbar orthosis on gait and sagittal alignment in patients with an osteoporotic vertebral compression fracture
	127	Abe, T.; Shibao, Y.; Takeuchi, Y.; Mataki, Y.; Amano, K.; Hioki, S.; Miura, K.; Noguchi, H.; Funayama, T.; Koda, M.; Yamazaki, M.	2018	Initial hospitalization with rigorous bed rest followed by bracing and rehabilitation as an option of conservative treatment for osteoporotic vertebral fractures in elderly patients: a pilot one arm safety and feasibility study
	128	Schiller, J.; Korallus, C.; Bethge, M.; Karst, M.; Schmalhofer, M. L.; Gutenbrunner, C.; Fink, M. G.	2016	Effects of acupuncture on quality of life and pain in patients with osteoporosis-a pilot randomized controlled trial
	129	Bouxsein, M.L.; Chen, P.; Glass, E.V.; Kallmes, D.F.; Delmas, P.D.; Mitlak, B.H.	2009	Teriparatide and raloxifene reduce the risk of new adjacent vertebral fractures in postmenopausal women with osteoporosis: Results from two randomized controlled trials
	130	Bornemann, R.; Kabir, K.; Otten, L.A.; Deml, M.; Koch, E.M.W.; Wirtz, D.C.; Pflugmacher, R.	2012	Radiofrequency Kyphoplasty - An Innovative method for the treatment of vertebral compression fractures - Comparison with conservative treatment
	131	Liu, Q.; Cao, J.; Kong, J.J.	2019	Clinical effect of balloon kyphoplasty in elderly patients with multiple osteoporotic vertebral fracture
	132	Abdalla, M. A.; Rodrigues, R.; Ulbricht, C.	2023	Vertebral Augmentation for Painful Type 4 Osteoporotic Compression Fractures: A Comparative Study
	133	Li, H.; Gu, Y.-F.; Li, Y.-D.; Wu, C.-G.; Li, M.-H.; Song, H.-M.	2012	Comparison of percutaneous vertebroplasty with conservative measures for chronic painful osteoporotic spinal fractures: A nonrandomized prospective trial

© 2024 Alimy AR et al. *JAMA Network Open.*

eTable 5: Characteristics of the included studies

Author (year) and country	Design	Study population				Intervention/Control		Finding
		N	Mean age (years)	Fracture recency	Diagnostic verification of fracture recency	Intervention	Control	
**Armingeat (2006) France**	RCT	32	70.8	<21 days	Pain<br>X-ray<br>MRI or scintigraphy	Pamidronate (30 mg, IV for three consecutive days)	Isotonic saline (500 ml, IV for three consecutive days)	Pamidronate was more efficacious than placebo regarding standing pain at day 7 and day 30.
**Endo (2017) Japan**	RCT	228	77.3	<2 weeks	Pain<br>X-ray	Elcatonin (20 units, IM injection, weekly)	NSAIDs (loxoprofen sodium [60mg, P.O. up to 3x daily], diclofenac sodium [25 mg, P.O., 3x daily], etodolac: [200 mg, P.O., 2x daily], lornoxicam [4 mg, P.O., 3x daily] or zaltoprofen [80 mg, P.O., 3x daily]	Calcitonin was more efficacious than NSAIDs regarding acute pain and quality of life at weeks 4 and 6.
**Hoshino (2013) Japan**	PCS	362	76.3	NA	Pain<br>X-ray<br>MRI	Braces<br>Hospitalization<br>Bisphosphonates<br>Painkillers	No Brace	No differences between braces, bisphosphonates, and analgesics after 6 months.
**Ikeda (2020) Japan**	RCT	96	78.1 vs 80.3	<1 week	Pain<br>X-ray	Teriparatide (56.5 µg, SC, weekly)	Alendronate (35 mg, P.O., weekly)	Teriparatide was more efficacious in increasing quality of life after 12 weeks compared to alendronate.
**Kataoka (2023) Japan**	RCT	65	81.1 vs 81.7	NA	Pain<br>X-ray<br>MRI	Pain management program	Usual Rehabilitation program	A rehabilitation program combined with pain management that targeted pain perception and activity avoidance was more efficacious regarding pain reduction and physical activity level compared to the usual rehabilitation program after two weeks and at discharge.

© 2024 Alimy AR et al. *JAMA Network Open.*

**Kato (2019) Japan**	RCT	382	76 vs 75.5	<4 weeks	Pain<br>X-ray<br>MRI	Rigid Brace	Soft Brace	No differences regarding pain reduction and quality of life between rigid and soft braces after 12 weeks of treatment.
**Kim (2014) Republic of Korea**	RCT	60	72.25 vs 66.75	Within 3 days	Pain<br>X-ray<br>MRI	Rigid Brace or<br>Soft Brace	No Brace	No differences regarding pain reduction, quality of life and disability between rigid braces, soft braces, and no braces.
**Ko (2021) Republic of Korea**	PCS	130	74.75 (76.09 vs 74.05)	NA	Pain<br>X-ray<br>MRI/CT	Vitamin D Supplementation (300,000 IU or 100,000 IU SC, followed by oral supplementation to retain appropiate serum levels)	No Supplementation	Vitamin D supplementation of patients with deficiency of 25(OH)D did not prove to be more efficacious regarding pain and quality of life compared to the control group after 3, 6 and 12 months.
**Laroche (2006) France**	RCT	27	71.5 (74 vs 68.8)	<4 months (Mean duration of pain at inclusion was 41.6 ± 38.4 days)	Pain<br>scintigraphy	Synthetic human calcitonin (1.5 mg in 500 mL saline, IV infusion, for 4h)	Pamidronate (1 mg/kg in 500 mL saline) IV infusion, for 4h)	No differences between calcitonin and pamidronate regarding pain and disability after 1 month of treatment.
**Li (2014) Hong Kong**	RCT	51	82 vs 81	NA	Pain<br>X-ray	Semirigid brace	Soft brace	No differences between Semirigid brace and soft brace regarding pain and function.
**Lyritis (1997) Greece**	RCT	100	76 (male) 71 (female)	<5 days	Pain<br>X-ray	Salmon calcitonin (200 IU, nasal spray, daily)	Identical placebo (nasal spray, daily)	Calcitonin was more efficacious than placebo regarding pain while resting and during activity after weeks 1, 2, 3 and 4.
**Lyritis (1999) Greece**	RCT	40	71	<5 days	Pain<br>X-ray	Salmon calcitonin (200 IU, suppository, daily)	Placebo (suppository, daily)	Calcitonin was more efficacious than placebo regarding pain while resting and during activity after weeks 1, 2, 3 and 4.
**Meccariello (2016) Italy**	PCS	140	82.3 (81.9 vs 82.8)	NA	Pain<br>X-ray<br>CT	3-point brace	Semirigid brace<br>dynamic corset	Semirigid brace was more efficacious in reducing pain and improving functional outcome and quality of life after 3 and 6 months, compared to patients treated with a 3-point brace.

© 2024 Alimy AR et al. *JAMA Network Open.*

**Rahman (2002) Bangladesh**	RCT	32	62.12 (61.21 vs 63.17)	<2weeks	Pain X-ray	Ipriflavone (200 mg, P.O., 3 times daily	Placebo (P.O., 3 times daily)	Ipriflavone was more efficacious in reducing pain than placebo after 3 months of treatment.
**Shigenobu (2019) Japan**	RCT	43	78.1 (80.2 vs 75.6)	NA (“fresh”)	MRI	Alendronate (35 mg, P.O., weekly) or risedronate, (17.5 mg, P.O., weekly) or risedronate (75 mg, P.O., monthly)	Teriparatide (dosage NA, SC, weekly)	Teriparatide and Bisphosphonates were both efficacious in reducing pain. Nevertheless, Teriparatide resulted in a higher quality of life compared to bisphosphonates after 24 weeks
**Shim (2021) Korea**	RCT	14	70.6 vs 76.3	<6 weeks (“recent”)	MRI	WJ-MSCs (intra-medullary [4 × 10^7 cells] injection into the fractured vertebra at baseline and intravenous [2 × 10^8 cells] injection after 1 week) and teriparatide (20 μg, SC, daily)	Teriparatide (20 μg, SC, daily)	Combined treatments of Wharton's jelly-derived MSCs and teriparatide was more efficacious regarding pain relief and quality of life compared to solely teriparatide at 1-year follow-up.
**Tanaka (2017a) Japan**	RCT	107	74.7 (74.3 vs 75)	<2 weeks	Pain X-ray MRI	Elcatonin (20 units, IM, weekly)	NSAIDs (Etodolac [200 mg, P.O., twice daily]) and a vitamin D analogue (0.5 μg, P.O., daily)	Elcatonin was more efficacious than NSAIDs regarding pain relief, quality of life and activities of daily living after 1 month and later.
**Tanaka (2017b) Japan**	RCT	51	75.5 and 78.1 vs 72.6	<2 weeks	Pain X-ray MRI	Elcatonin (20 units, IM injection, weekly) or combined elcatonin (20 units, IM injection) + minodronic acid hydrate (1 mg, P.O., daily)	Minodronic acid hydrate (1 mg, P.O., daily)	Elcatonin and a combination of elcatonin and minodronic acid hydrate was more efficacious regarding pain relief than sole use of minodronic acid hydrate alone after 4 weeks.

© 2024 Alimy AR et al. *JAMA Network Open.*

**Tsuchie (2016) Japan**	PCS	34	82 (81.9 and 83 vs 80.3)	NA ("fresh")	MRI	Daily teriparatide (20 µg, SC, daily) or<br>weekly teriparatide (56.5 µg, SC, weekly)	Risedronate (17.5 mg, P.O., weekly)	Both daily and weekly teriparatide were more efficacious than risedronate regarding pain at 8 and 12 weeks after the initial visit.
**Vorsanger (2013) USA**	RCT	108	69.5 (69.8 and 69.3 vs 69.6)	NA (X-ray within 3 months demonstrating at least one VCF)	Pain<br>X-ray	Tapentadol (50 mg, then 50 or 75 mg, IR, P.O., every 4 to 6 hours) or Oxycodone (5 mg, then 5 or 10 mg IR, P.O., every 4 to 6 hours)	Placebo (every 4 to 6 hours)	In this prematurely terminated study both tapentadol and oxycodone showed tendencies towards more efficacious total pain relief and pain intensity differences compared to placebo. There were no differences between tapentadol and oxycodone regarding pain relief.

Abbreviations: IR = Immediate-Release, IV = Intravenous, MRI = Magnetic Resonance Imaging, NSAID = Non-steroidal anti-inflammatory drug, P.O. = Per Os (orally), PCS = prospective comparative study, RCT = randomized controlled trial, SC = Subcutaneous, VCF = Vertebral Compression Fracture, WJ-MSC = Wharton's jelly-derived mesenchymal stem cell

© 2024 Alimy AR et al. *JAMA Network Open.*

eTable 6: Study quality of the included prospective, comparative studies

Study quality rating based on the Newcastle-Ottawa-Scale.

**Study**	**Study quality**
Ko *et al.* 2021	Good
Hoshino *et al.* 2013	Good
Tsuchie *et al.* 2016	Good
Meccariello *et al.* 2016	Good

© 2024 Alimy AR et al. *JAMA Network Open.*

eTable 7: Estimates of effects and GRADE quality ratings for comparison of different pharmacological interventions for short-term pain during activity

	Direct evidence	Certainty of evidence	Indirect evidence	Certainty of evidence	Network meta-analysis	Certainty of evidence
Bisphosphonate vs Calcitonin			3.95 [0.54 to 7.36]	Low §, ‡	3.95 [0.54 to 7.36]	Low §, ‡
Bisphosphonate vs NSAID			3.03 [-1.32 to 7.37]	Very low §, ‡, †	3.03 [-1.32 to 7.37]	Very low §, ‡, †
Bisphosphonate vs Placebo	-0.91 [-3.68 to 1.85]	Low ‡, †			-0.91 [-3.68 to 1.85]	Low ‡, †
Bisphosphonate vs Teriparatide	0.10 [-2.60 to 2.79]	Very low §, ‡, †			0.10 [-2.60 to 2.79]	Very low §, ‡, †
Calcitonin vs NSAID	-0.92 [-3.62 to 1.77]	Very low §, ‡, †			-0.92 [-3.62 to 1.77]	Very low §, ‡, †
Calcitonin vs Placebo	**-4.86 [-6.87 to -2.86]**	Low §, ‡			**-4.86 [-6.87 to -2.86]**	Low §, ‡
Calcitonin vs Teriparatide			-3.85 [-8.20 to 0.50]	Very low §, ‡, †	-3.85 [-8.20 to 0.50]	Very low §, ‡, †
NSAID vs Placebo			-3.94 [-7.30 to -0.58]	Low §, ‡	-3.94 [-7.30 to -0.58]	Low §, †
NSAID vs Teriparatide			-2.93 [-8.04 to 2.19]	Very low §, ‡, †	-2.93 [-8.04 to 2.19]	Very low §, ‡, †
Teriparatide vs Placebo			-1.01 [-4.87 to 2.85]	Very low §, ‡, †	-1.01 [-4.87 to 2.85]	Very low §, ‡, †

†Imprecision, ‡ Indirectness, §Risk of bias

© 2024 Alimy AR et al. *JAMA Network Open.*

eTable 8: Adverse events of the included studies.

Study	Intervention	Control	Complications of the intervention group	Complications of the control group
Armingeat (2006)	Pamidronate (30 mg, IV for three consecutive days)	Isotonic saline (500 ml, IV for three consecutive days)	Fever and transient muscle pain, (n=2), 12.5%	Epileptic seizure, (n=1), 6.7%
Endo (2017)	Elcatonin (20 units, IM injection, weekly)	NSAIDs (loxoprofen sodium [60mg, P.O. up to 3x daily], diclofenac sodium [25 mg, P.O., 3x daily], etodolac: [200 mg, P.O., 2x daily], lornoxicam [4 mg, P.O., 3x daily] or zaltoprofen [80 mg, P.O., 3x daily]	None reported	Upper GI disorder, (n=1), 0.88%, Drug eruption, (n=2), 1.75%
Hoshino (2013)	Braces Hospitalization Bisphosphonates Painkillers	No brace	NA due to the multivariate analysis in the study	NA due to the multivariate analysis in the study
Ikeda (2020)	Teriparatide (56.5 µg, SC, weekly)	Alendronate (35 mg, P.O., weekly)	Delayed union, (n=16), 33.3%	Delayed Union, (n=19), 39.6%
Kataoka (2023)	Pain management program	Usual Rehabilitation program	Acute cholecystitis (n=1), 3.0% Repeated vertebral fractures, (n=1), 3.0%	Pneumonia, (n=1), 3.1%, Acute heart failure, (n=1), 3.1%
Kato (2019)	Rigid Brace	Soft Brace	New vertebral fractures, (n=7), 4.96%	New vertebral fractures, (n=9), 6.3%, Secondary operation, (n=2), 1.40%
Kim (2014)	Rigid Brace or Soft Brace	No Brace	None reported	None reported
Ko (2021)	Vitamin D Supplementation (300,000 IU or 100,000 IU SC, followed by oral supplementation to retain appropiate serum levels)	No Supplementation	None reported	None reported
Laroche (2006)	Synthetic human calcitonin (1.5 mg in 500 mL saline, IV infusion, for 4h)	Pamidronate (1 mg/kg in 500 mL saline) IV infusion, for 4h)	Hot flushes, or nausea without vomiting (n=5), 38.5%	Muscle pain (n=1), 7.1%
Li (2014)	Semirigid brace	Soft brace	None reported	None reported
Lyritis (1997)	Salmon calcitonin (200 IU, nasal spray, daily)	Identical placebo (nasal spray, daily)†	Mild symptoms, mainly headache (n=6), 12%	None reported
Lyritis (1999)	Salmon calcitonin (200 IU, suppository, daily)	Placebo (suppository, daily)	Mild dizziness, (n=7), 36.8% Mild enteric disturbances, (n=11), 57.9%	Mild dizziness, (n=1), 6.3%, Mild enteric disturbances, (n=7), 43.8%

© 2024 Alimy AR et al. *JAMA Network Open.*

Meccariello (2016)	3-point brace	Semirigid brace	Gastric ulcer, (n=6), 8.3%,<br>Decubitus, (n=8), 11.1%,<br>Inguinal hernia, (n=4), 5.6%,<br>Pulmonary disease, (n=4), 5.6%,<br>Refractures, (n=6), 8.3%	Gastric ulcer, (n=6), 8.8%,<br>Refractures, (n=2), 2.9%
Rahman (2002)	Ipriflavone (200 mg, P.O., 3 times daily)	Placebo (P.O., 3 times daily)	Gastrointestinal disturbances, (n=6), 42.9%,<br>Headache, (n=2), 14.3%,<br>Vertigo, (n=1), 7.1%,<br>Insomnia, (n=1), 7.1%	Gastrointestinal disturbances, (n=5), 41.7%,<br>Headache, (n=1), 8.3%,<br>Vertigo, (n=1), 8.3%,<br>Insomnia, (n=1), 8.3%
Shigenobu (2019)	Alendronate (35 mg, P.O., weekly) or risedronate, (17.5 mg, P.O., weekly) or risedronate (75 mg, P.O., monthly)	Teriparatide (dosage NA, SC, weekly)	medical complication, (not further specified) (n=1), 4.2%	Nausea or vomiting, (n=3), 15.8%
Shim (2021)	WJ-MSCs (intra-medullary [4 × 10^7 cells] injection into the fractured vertebra at baseline and intravenous [2 × 10^8 cells] injection after 1 week) and teriparatide (20 µg, SC, daily)	Teriparatide (20 µg, SC, daily)	UTI, (n=1), 10%,<br>Itching, (n=1), 10%,<br>Pulmonary Embolism, (n=1), 10%,<br>Incidental finding tumor, (n=1), 10%	Nausea, (n=1), 10%,<br>Vomiting, (n=1), 10%,<br>Dizziness, (n=1), 10%
Tanaka (2017a)	Elcatonin (20 units, IM, weekly)	NSAIDs (Etodolac [200 mg, P.O., twice daily]) and a vitamin D analogue (0.5 μg, P.O., daily)	None reported	None reported
Tanaka (2017b)	Elcatonin (20 units, IM injection, weekly) or combined elcatonin (20 units, IM injection) + minodronic acid hydrate (1 mg, P.O., daily)	Minodronic acid hydrate (1 mg, P.O., daily)	None reported	None reported
Tsuchie (2016)	Daily teriparatide (20 µg, SC, daily) or weekly teriparatide (56.5 µg, SC, weekly)	Risedronate (17.5 mg, P.O., weekly)	Cleft formation, (n=1), 5%	Cleft formation, (n=6), 42.9%
Vorsanger (2013)	Tapentadol (50 mg, then 50 or 75 mg, IR, P.O., every 4 to 6 hours) or Oxycodone (5 mg, then 5 or 10 mg IR, P.O., every 4 to 6 hours)	Placebo (every 4 to 6 hours)	Tapentadol:<br>Nausea, (n=15), 34.1%,<br>Vomiting, (n=12), 27.3%,<br>Constipation, (n=1), 2.3%,<br>Anorexia, (n=1), 2.3%,<br>Dizziness, (n=8), 18.2%,<br>Somnolence, (n=4), 9.1%,<br>Oxycodone:<br>Nausea, (n=19), 44.2%,<br>Vomiting, (n=19), 44.2%,<br>Constipation, (n=3), 7.0%,<br>Anorexia, (n=3), 7.0%,<br>Dizziness, (n=7), 16.3%,<br>Somnolence, (n=5), 11.6%,<br>Headache, (n=2), 4.7%	Nausea, (n=1), 4.8%,<br>Vomiting, (n=4), 19.9%,<br>Constipation, (n=1), 4.8%,<br>Anorexia, (n=1), 4.8%,<br>Somnolence, (n=2), 9.5%,

© 2024 Alimy AR et al. *JAMA Network Open.*

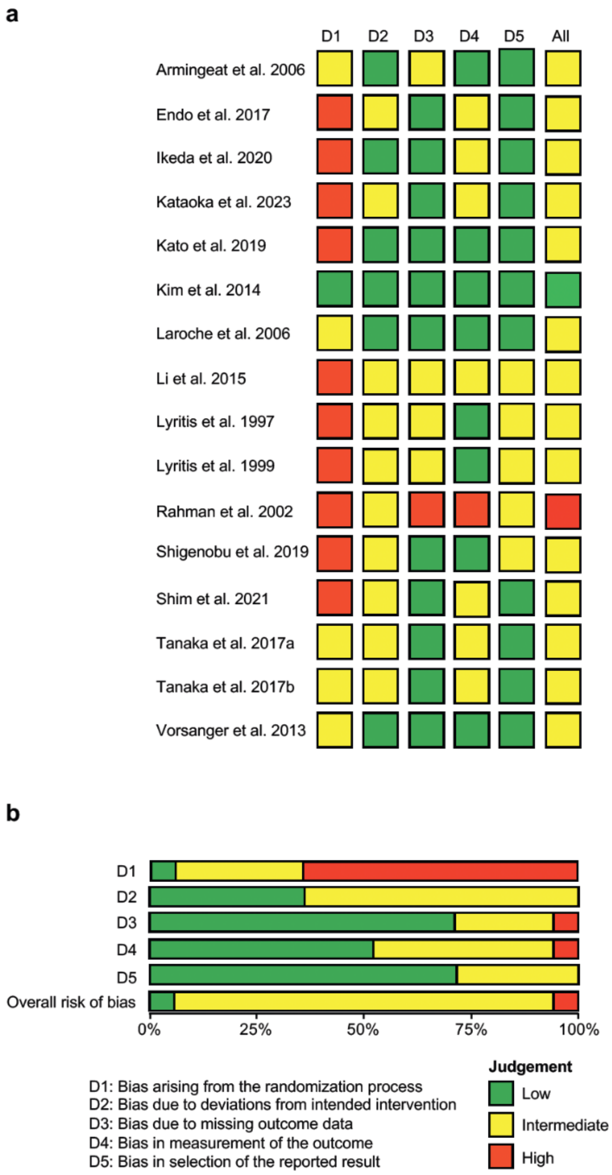

eFigure 1: Risk of bias assessment

Risk of bias assessment for the included studies using the second version of the Cochrane tool for assessing the risk of bias in randomized trials (RoB2). A) Judgements were made regarding each risk of bias domain in all included randomized controlled studies. B) The Assessment is visualized as percentages across all studies.

© 2024 Alimy AR et al. *JAMA Network Open.*

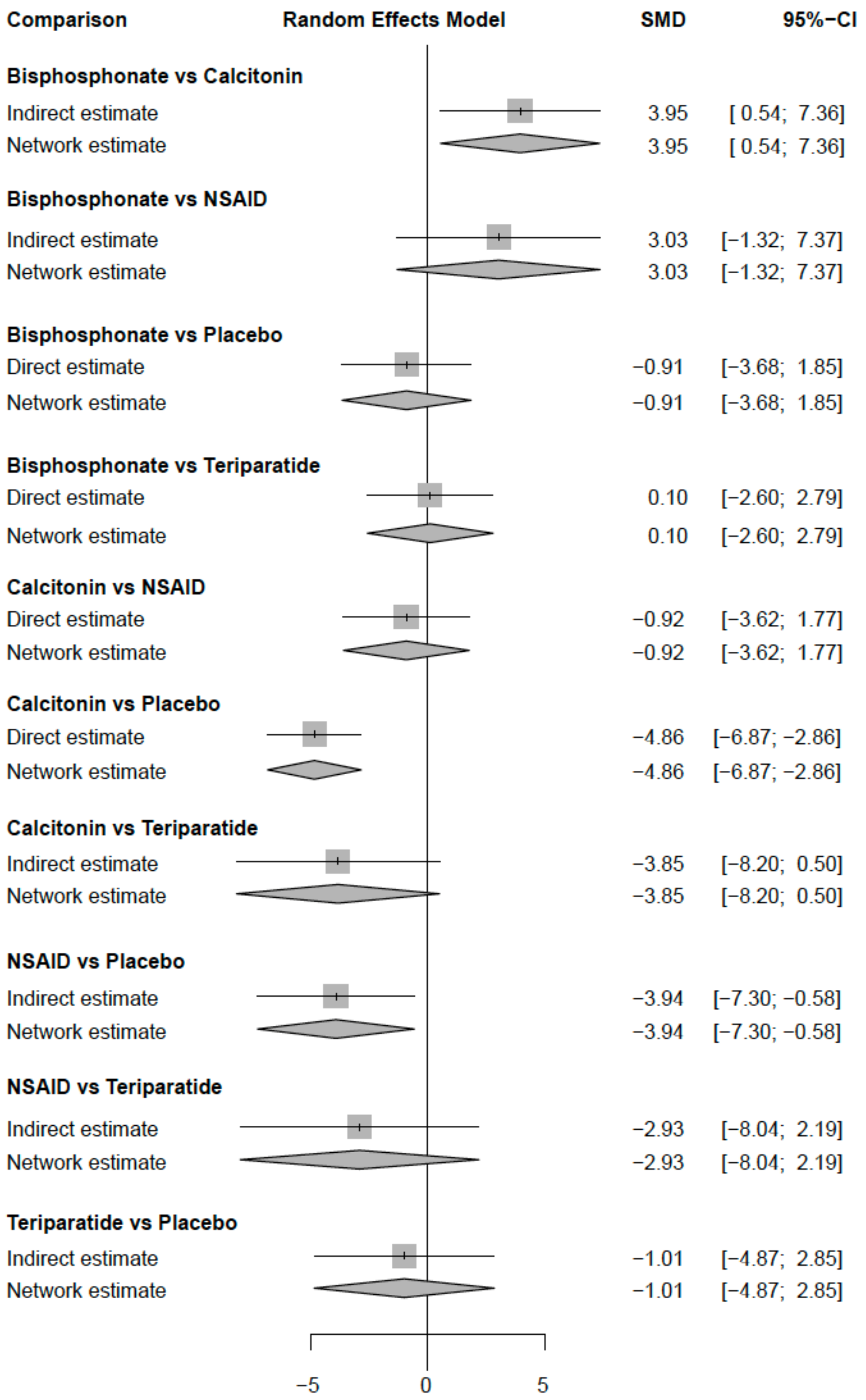

eFigure 2: Node splitting: Short-term pain during activity

Forest plot for short-term pain management during rest illustrating the results of node splitting, comparing direct, indirect, and network estimates. Data are presented as the standardized mean difference (SMD) [95% CI] for short-term pain during activity. Values below 0 indicate that the treatment mentioned first (before the "vs.") is favored, while values above 0 indicate that the treatment mentioned (after the "vs.") is favored regarding pain outcomes. SMD = standardized mean difference; CI = confidence interval; NSAID = Non-steroidal anti-inflammatory drug.

© 2024 Alimy AR et al. *JAMA Network Open.*

Outcome	Intervention	P-Score
Short-term: Pain (Activity)	Calcitonin	0.92
	NSAID	0.76
	Teriparatide	0.35
	Bisphosphonate	0.33
	Placebo	0.14

eFigure 3: P-score ranking: Short-term pain during activity

Comparative ranking of pharmacological interventions for short-term pain during activity using P-scores. Treatments are ranked using P-scores, which enables ranking treatment on a continuous scale from 0 to 1, where higher scores indicate that a treatment is better than the competing treatments.

© 2024 Alimy AR et al. *JAMA Network Open.*

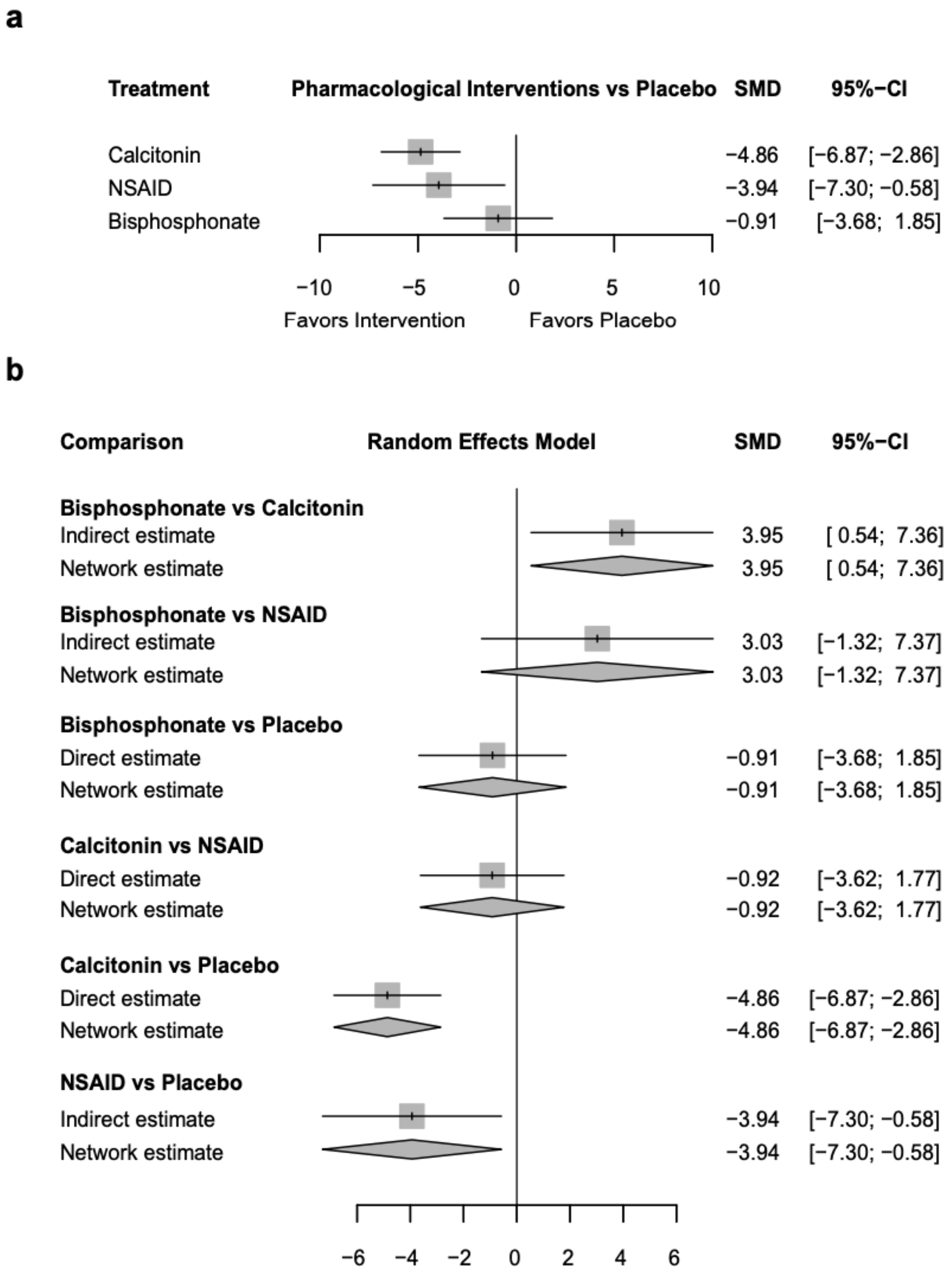

eFigure 4: Sensitivity analyses: Short-term pain during activity (walking and rising up).

A) Network meta-analysis short-term pain management during activity including only walking and rising up from a lying position, comparing pharmacological interventions with placebo. B) Forest plot for short-term pain management during activity, including only walking and rising up from a lying position, illustrating the results of node splitting, comparing direct, indirect, and network estimates for the sensitivity analysis. Data are presented as the standardized mean difference (SMD) [95% CI] for sensitivity analyses of short-term pain during activity. Values below 0 indicate that the treatment mentioned first (before the "vs.") is favored, while values above 0 indicate that the treatment mentioned (after the "vs.") is favored regarding pain outcomes.

© 2024 Alimy AR et al. *JAMA Network Open.*

a

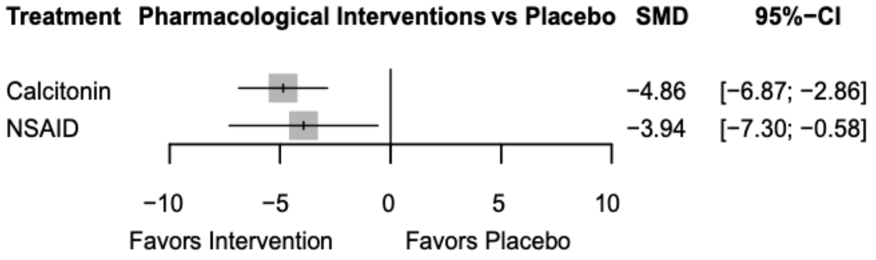

b

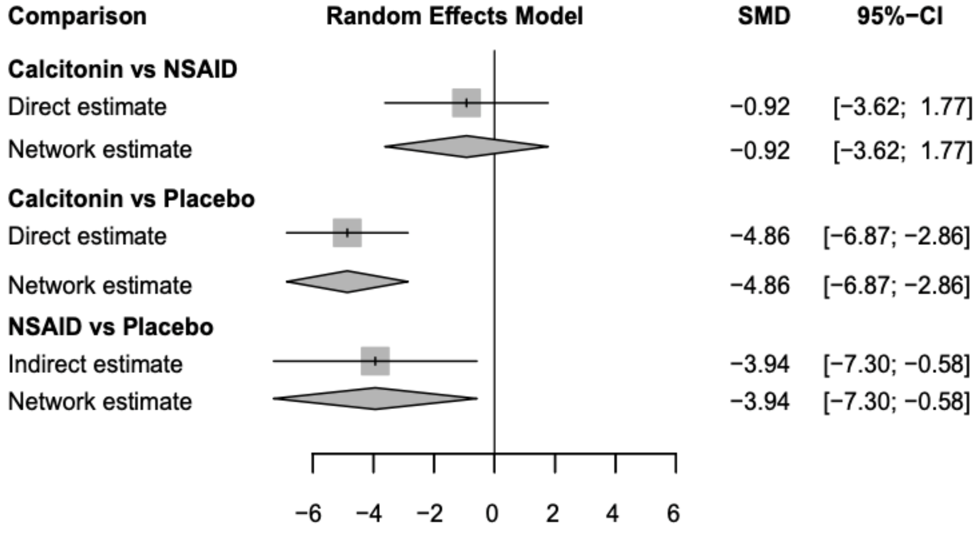

eFigure 5: Sensitivity analyses: Short-term pain during activity (only walking).

a) Network meta-analysis short-term pain management during activity including only, comparing pharmacological interventions with placebo. b) Forest plot for short-term pain management during activity, including only walking, illustrating the results of node splitting, comparing direct, indirect, and network estimates for the sensitivity analysis. Data are presented as the standardized mean difference (SMD) [95% CI] for sensitivity analyses of short-term pain during activity. Values below 0 indicate that the treatment mentioned first (before the "vs.") is favored, while values above 0 indicate that the treatment mentioned (after the "vs.") is favored regarding pain outcomes.

© 2024 Alimy AR et al. *JAMA Network Open.*

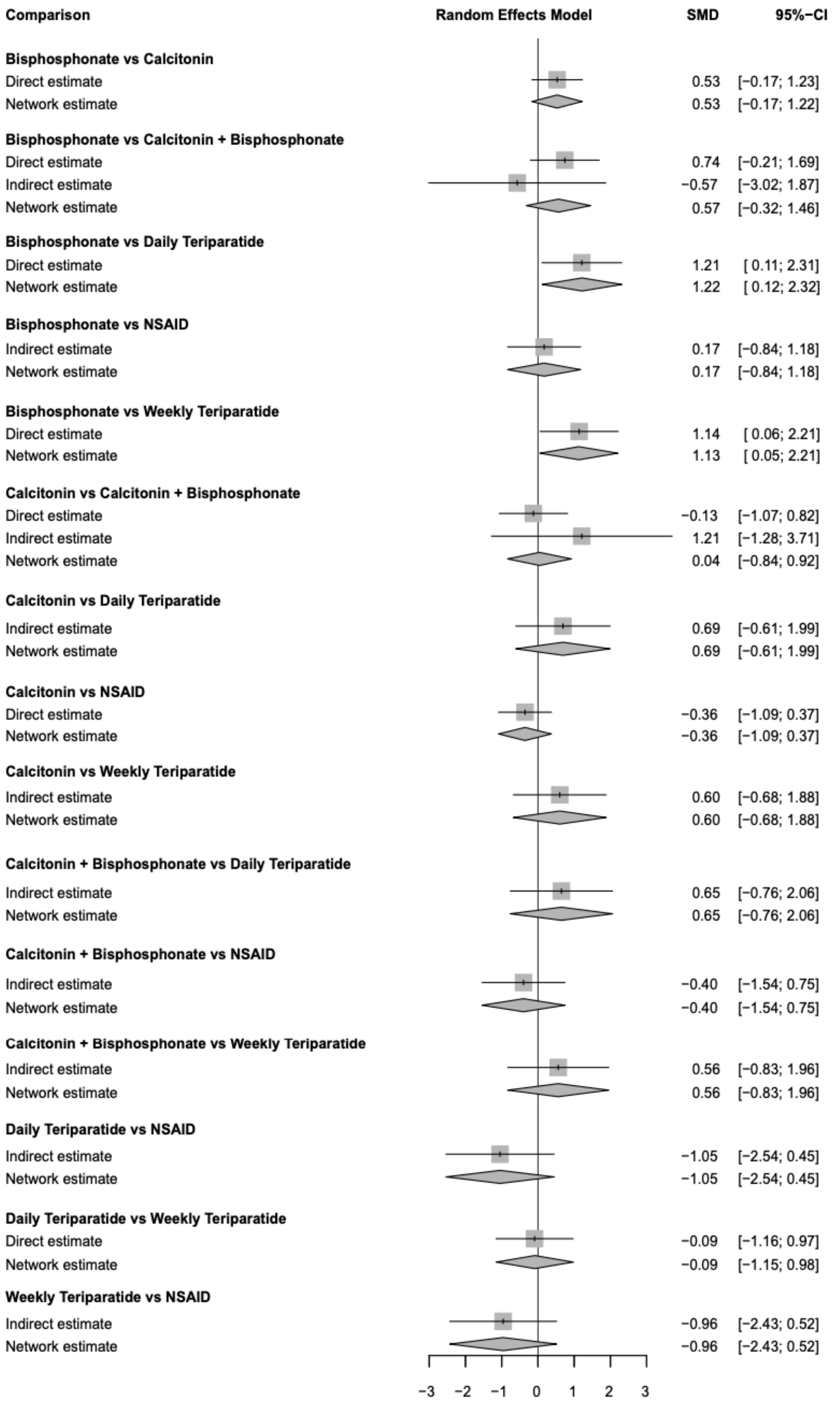

eFigure 6: Node splitting: Pharmacological interventions (long-term pain).

Forest plot for long-term pain management of illustrating the results of node splitting, comparing direct, indirect, and network estimates SMD = standardized mean difference; CI = confidence interval; NSAID = Non-steroidal anti-inflammatory drug.

© 2024 Alimy AR et al. *JAMA Network Open.*

Outcome	Intervention	P-Score
Pain (long-term) Pharmacological Interventions	Daily Teriparatide	0.83
	Weekly Teriparatide	0.78
	Calcitonin and Bisphosphonate	0.52
	Calcitonin	0.51
	NSAID	0.25
	Bisphosphonate	0.12

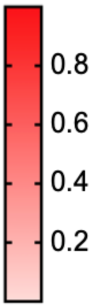

eFigure 7: P-score ranking: Pharmacological interventions for long-term pain

Comparative ranking of pharmacological interventions for long-term pain using P-scores. Treatments are ranked using P-scores, which enables ranking treatment on a continuous scale from 0 to 1, where higher scores indicate that a treatment is better than the competing treatments.

© 2024 Alimy AR et al. *JAMA Network Open.*

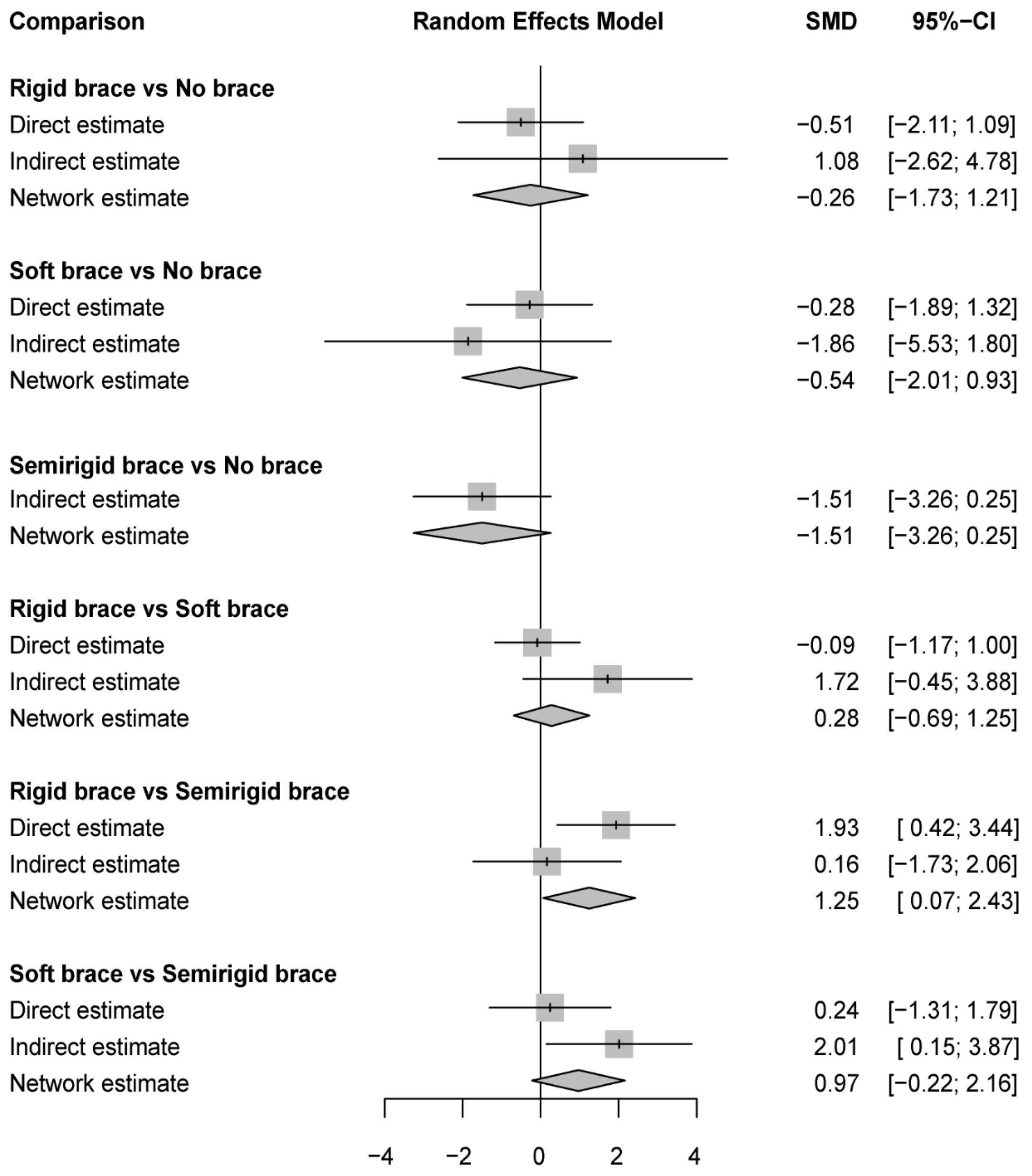

eFigure 8: Node splitting: Braces (long-term pain).

Forest plot for long-term pain management of illustrating the results of node splitting, comparing direct, indirect, and network estimates
SMD = standardized mean difference; CI = confidence interval.

© 2024 Alimy AR et al. *JAMA Network Open.*

Outcome	Intervention	P-Score
Pain (long-term)		
Braces	Semirigid brace	0.96
	Soft brace	0.51
	Rigid brace	0.31
	No brace	0.22

eFigure 9: P-score ranking: Braces for long-term pain

Comparative ranking of braces for long-term pain using P-scores. Treatments are ranked using P-scores, which enables ranking treatment on a continuous scale from 0 to 1, where higher scores indicate that a treatment is better than the competing treatments.

© 2024 Alimy AR et al. *JAMA Network Open.*

eReferences

1. Wan X, Wang W, Liu J, Tong T. Estimating the sample mean and standard deviation from the sample size, median, range and/or interquartile range. *BMC Med Res Methodol*. Dec 19 2014;14:135. doi:10.1186/1471-2288-14-135
2. PlotDigitizer. Accessed 05.03.2024), https://plotdigitizer.com/
3. Peterson J, Welch V, Losos M, Tugwell P. The Newcastle-Ottawa scale (NOS) for assessing the quality of nonrandomised studies in meta-analyses. *Ottawa: Ottawa Hospital Research Institute*. 2011;2(1):1-12.
4. Balduzzi S, Rücker G, Nikolakopoulou A, et al. netmeta: An R Package for Network Meta-Analysis Using Frequentist Methods. *Journal of Statistical Software*. Mar 2023;106(2):1-40. doi:10.18637/jss.v106.i02
5. Higgins JP, Thompson SG. Quantifying heterogeneity in a meta-analysis. *Stat Med*. Jun 15 2002;21(11):1539-58. doi:10.1002/sim.1186
6. Dias S, Welton NJ, Caldwell DM, Ades AE. Checking consistency in mixed treatment comparison meta-analysis. *Stat Med*. Mar 30 2010;29(7-8):932-44. doi:10.1002/sim.3767
7. Higgins JP, Green S. Cochrane handbook for systematic reviews of interventions. 2008;
8. Rucker G, Schwarzer G. Ranking treatments in frequentist network meta-analysis works without resampling methods. *BMC Med Res Methodol*. Jul 31 2015;15:58. doi:10.1186/s12874-015-0060-8
