# Supplementary material for: Conservative Treatments in the Management of Acute Painful Vertebral Compression Fractures: A Systematic Review and Network Meta-Analysis
Source: JAMA Netw Open. 2024 Sep 6;7(9):e2432041. doi: 10.1001/jamanetworkopen.2024.32041 (PMC11380106; doi:10.1001/jamanetworkopen.2024.32041)
Supplement: Supplement 2. — Data Sharing Statement [file jamanetwopen-e2432041-s002.pdf]

Data Sharing Statement

Alimy. Conservative Treatments in the Management of Acute Painful Vertebral Compression Fractures. *JAMA Netw Open*. Published September 06, 2024.
doi:10.1001/jamanetworkopen.2024.32041

Data
Data available: No

Additional Information
Explanation for why data not available: All data are available in the manuscript and in the source publications that were used for the data synthesis.
